# Recycling of High-Purity Lithium Metal from Waste Battery by Photoelectrochemical Extraction at Ultralow Overall Potential

**DOI:** 10.1007/s40820-025-01958-z

**Published:** 2026-01-14

**Authors:** Longfei Yang, Chao Huang, Yanhong Lyu, Dawei Chen, Aibin Huang, Jianyun Zheng

**Affiliations:** 1 State Key Laboratory of Chemo and Biosensing, College of Chemistry and Chemical Engineering, International Joint Lab of Energy Electrochemistry of the Ministry of Education, Hunan University, Changsha, 410082 People’s Republic of China; 2 College of Material Science and Engineering, Qingdao University of Science and Technology, Zhengzhou Road 53, Qingdao, 266042 People’s Republic of China; 3 State Key Laboratory of High Performance Ceramics and Superfine Microstructure, Shanghai Institute of Ceramics, Chinese Academy of Sciences, Shanghai, 200050 People’s Republic of China; 4 Center of Materials Science and Optoelectronics Engineering, University of Chinese Academy of Sciences, Beijing, 100049 People’s Republic of China; 5 Shenzhen Research Institute of Hunan University, Shenzhen, 518055 People’s Republic of China; 6 School of Physical and Chemistry, Hunan First Normal University, Changsha, 410205 People’s Republic of China

**Keywords:** Photoelectrochemical method, Lithium metals, Waste batteries, Ultralow-potential device, Selective and efficient extraction

## Abstract

To ease the scarcity of lithium (Li) resource and cut down on environmental pollution, an efficient, selective, inexpensive and sustainable Li recycling process from waste batteries is needed, which is yet to be achieved. Here, we report a low-potential photoelectrochemical (PEC) system that selectively and efficiently extracts Li metals from multi-cation electrolytes under 1 sun illumination. Based on the difference of redox potential, we can get rid of the disturbance of other cations (i.e., Fe, Co and Ni ions) by a bias-free PEC device to realize the extraction of high-purity Li metals on a coplanar Si-based photocathode-TiO_2_ photoanode tandem device at 2 V of applied bias (far less than the redox potentials of Li^+^/Li). In such system, the extraction rate of Li metals (purity > 99.5%) exceeds 1.35 g h^−1^ m^−2^ with 90% of Faradaic efficiency. Long-term experiments, different electrode/electrolyte tests, and various price assessments further demonstrate the stability, compatibility and economy of PEC extraction system, enabling a solar-driven pathway for the recycling of critical metal resources.

**Supplementary Information:**

The online version contains supplementary material available at 10.1007/s40820-025-01958-z.

## Highlights

- A low-potential photoelectrochemical (PEC) system was designed and used to selectively and efficiently extracts Li metals from multi-cation electrolytes under 1 sun illumination.
- A coplanar Si-based photocathode-TiO_2_ photoanode PEC device exhibited an acceptable extraction rate of ~1.38 g h^−1^ m^−2,^ an excellent FE of 90.7% and a high production purity of 99.5%.
- The designed PEC system also showed potential for purifying the waste electrolytes and recycling the other metals (i.e., Fe, Co, and Ni).

**Supplementary Information:**

The online version contains supplementary material available at 10.1007/s40820-025-01958-z.

## Graphical abstract

**Figure:**
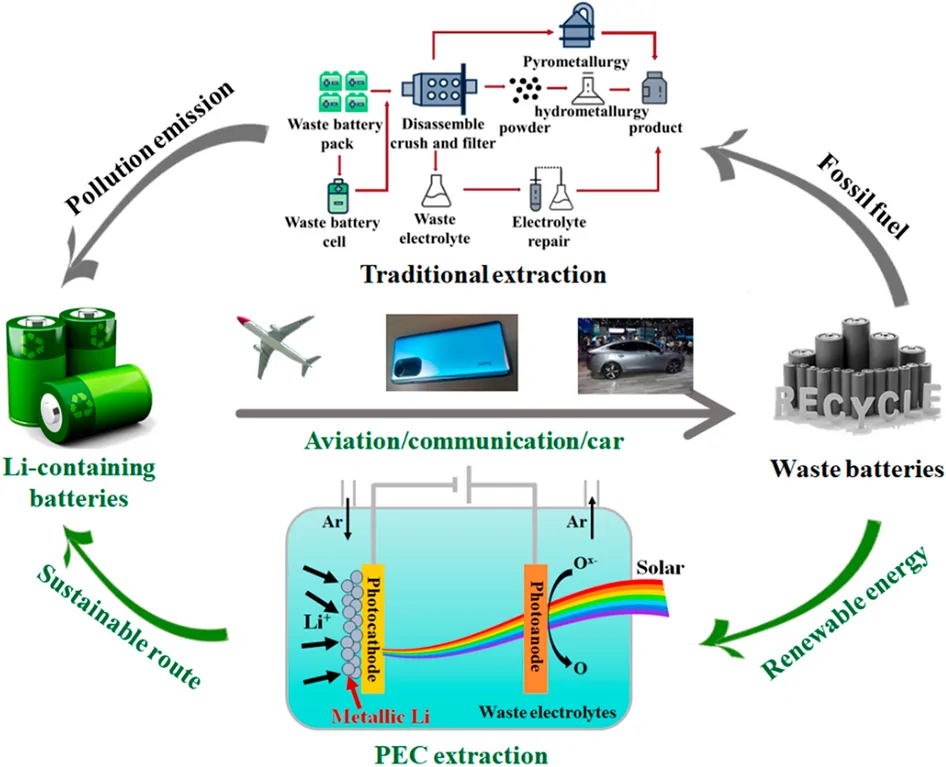

## Introduction

**Fig. 1:**
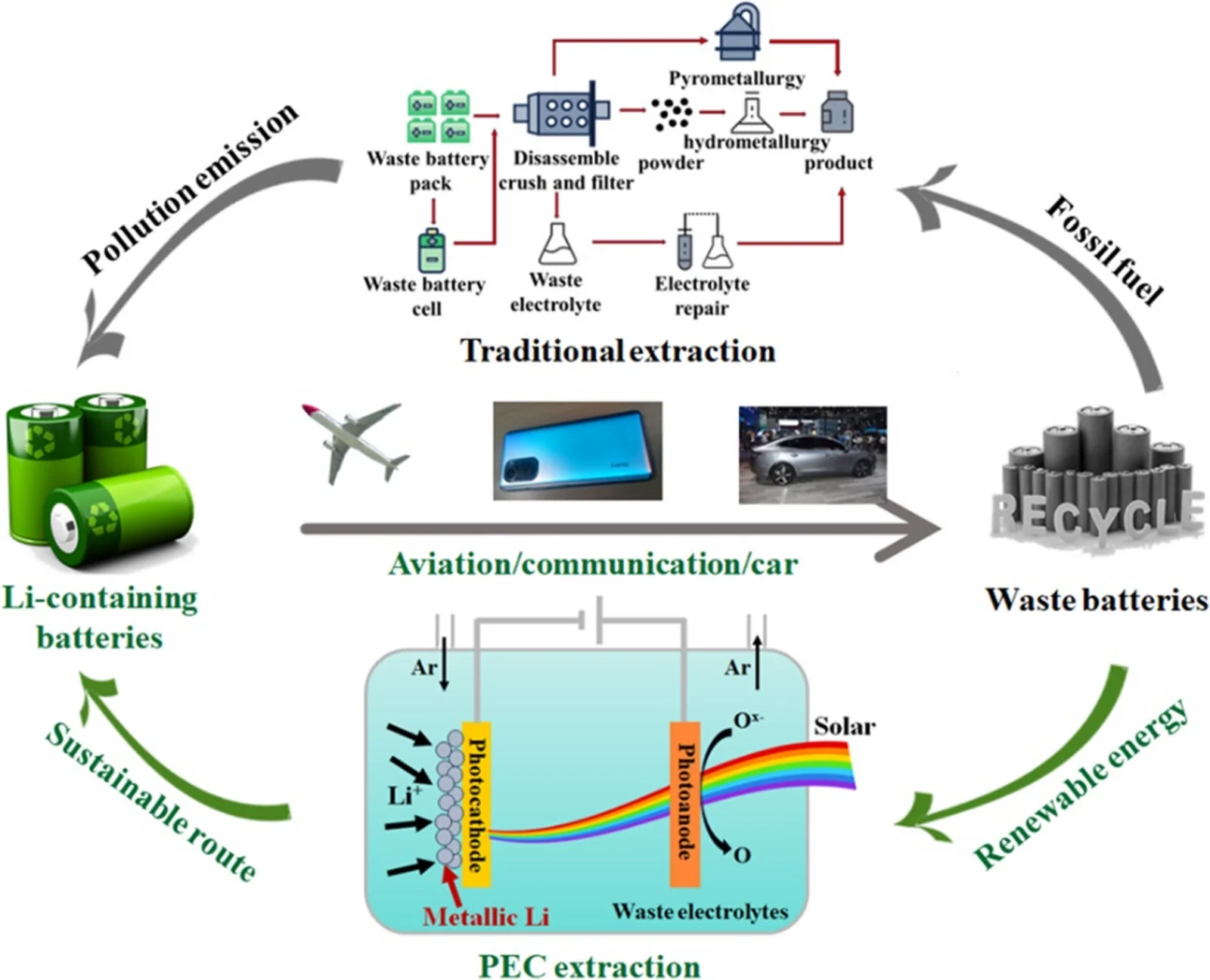
Recycling of Li. In a typical cycle of Li-based energy, Li-containing batteries are mostly used in renewable energy systems, portable electronic devices, and electric vehicles. After the expiration of service life, a mass of waste batteries can be produced with plentiful Li resources. In a traditional extraction process (like hydrometallurgical method, upper loop with gray arrow), waste batteries usually go through three steps of leaching, concentration/purification and recovery at high temperature (> 100 °C) in strongly acidic/basic solutions. In the possible PEC extraction process (lower loop with green arrow), Li ions in the electrolytes of waste batteries could be directly turned back to Li metals with a solar energy supply as a sustainable route

Lithium (Li) as a critical element in the sustainable energy industry chains has been identified as one of the global strategic mineral elements [1–3]. With the surging demand for portable electronic devices and electric vehicles (Fig. 1), the Li-containing battery market can be forecast to significantly expand in the next decade [4–6]. On basis of the current technology and practical requirement, each electric vehicle with the Li-containing batteries has to consume1.14 ~ 7.76 kg of Li at least [7]. Given that the total Li reserves on land are proved to be ~ 22 million tons, around 7.3 billion of electric vehicles are produced to exhaust these Li reserves, which can happen in 2080 with the continuous growth of Li consumption rate [8, 9]. To prepare for the disastrous effect to come, opening up the unconventional Li sources (such as seawater with > 230 billion tons of Li) and recycling the waste Li from useless products (such as waste batteries) are regarded as the most effective ways to develop the renewable energy systems [10, 11]. Compared with a lot of attention on the Li extraction from various brines [12–14], there are only certain studies on the recycle of Li from waste Li-containing batteries, especially directly obtaining metallic Li production. Currently, the service life of commercial Li-containing batteries (such as Li-ion battery) is ~ 10 years, and their structure can undergo irreversible changes leading to the failure of the batteries after ten hundreds of charge and discharge cycles [15]. With the ever-increasing demands of electric vehicle market, the number of retired Li-containing batteries increases sharply [16–18], which squanders away the limited Li sources on Earth and brings about a severe issue of environmental pollution [19]. If exploring a reliable and sustainable extracting method can effectively collect back and recycle the waste Li resources [20], this will offer a stable and green Li supply chain for satisfying the market demand and meanwhile reduce the environmental pollution (Fig. 1).

Taking Li-ion battery as an example, the overall proportion of valuable metals in a battery is 2~ 4 times higher than the metal content in natural ores and minerals [21]. Thereinto, Li, iron (Fe), cobalt (Co) and nickel (Ni) account for 2% ~ 5%, 3% ~ 10%, 5% ~ 20%, and 5% ~ 12%, respectively [22–24]. From an environmental perspective, the discarded Li-ion batteries with many metallic ions and organic compounds are directly harm the living environment of human beings. In contrast, from an economic point of view, the recycle and reuse of valuable metals (i.e., Li) from waste batteries by green technology can reduce the manufacturing and disposal costs of the batteries. According to the calculations by Tarascon et al. [25], 1 ton of Li can be extracted from ~ 28 tons of waste Li-ion batteries, which is a significant advantage over extracting Li from minerals (1 ton of Li/250 tons of minerals) or brines (1 ton of Li/750 tons of brines). Various Li extraction technologies have made attempts to effectively obtain the high-purity Li products, which can be grouped into the traditional and direct (i.e., additive-free) Li extraction methods [26]. Owing to the use of toxic chemicals, high energy/material consumption, undesirable by-product sludges and long operation time, the Li from waste batteries is difficult to be extracted by the traditional methods, mainly including solvent extraction, chemical precipitation, and evaporation concentration [27–29]. In recent years, considerable attention has been drawn to direct Li extraction methods as promising alternatives to traditional methods because of their higher efficiency and better environmental compatibility [30–32].

Electrochemical ion pumping as a typical direct extraction technology shows high selectivity of Li ions by the intercalation cathodes (i.e., LiCoO_2_ [33] and LiFePO_4_ [34]) with inherent preference for Li over other cations. But, low current density for inhibiting the co-intercalation of impurities and necessary post-treatment steps for refreshing the intercalated cathode limit the large-scale application of this technology [35]. In addition, as the next generation of extraction technology, although membrane-based separation approach (i.e., electrodialysis and nanofiltration) features many advantages including considerable yield rate, low chemical emission and continuous operation [36, 37], it usually suffers from poor selectivity toward Li, especially Li/Na and Li/Mg separation [38, 39]. Clearly, the extracted products by all the aforementioned techniques are Li ions dissolved in water, and further processing steps are carried out to obtain Li metals or solid Li compounds. The Li metals are widely used as the anode materials in Li-air and Li-sulfur (S) batteries and as the raw materials to synthesize the various solid Li-based compounds. Several pioneering works have been committed to exploring the extraction of Li metals from the Li-containing solution. In 2018, an electrolysis process has been developed to produce the Li metals with an acceptable selectivity from seawater at a high applied potential over 4 V, meaning high energy consumption in this process [40]. Very recently, Zheng and co-workers have utilized the solar-driven photoelectrochemical (PEC) methods (Fig. 1) to obtain the Li metals from the electrolytes of the Li-containing battery at ~ 0.1 V versus (vs.) Li^+^/Li redox couple (the standard redox potential of Li^+^/Li is −3.04 V vs. standard hydrogen electrode, SHE) [41]. Despite the high extraction rate, excellent Faradaic efficiency (FE) and effective solar utilization, this work is only an initial attempt to recycle the Li metals from the single-cation electrolytes, which is different from the multi-cation electrolytes of practical waste batteries (i.e., Li, Fe, Co and Ni). Considering that PEC method can simultaneously realize the recovery of waste Li resources and the conversion of solar energy, developing further PEC extraction of Li metals from multi-cation electrolytes at lower applied potential can push forward the practical process of valuable resource recycle from waste batteries. To further improve the PEC extraction performance of Li metals, the exploration of cocatalyst materials for reducing the reaction energy barriers is necessary. In fact, in addition to tungsten oxide (WO_3_) cocatalyst layer reported in our previous work [41], titanium oxide (TiO_2_) and molybdenum oxide (MoO_3_) as classic electrochromic materials can be promising candidates for facilitating the insertion of Li^+^ and concentrating the Li^+^ at the surface. However, the work mechanism of these materials for PEC Li extraction is unclear.

In this article, we leverage an untapped difference of redox potential between various metal cations (i.e., Li^+^/Li of −3.04 V vs. SHE, Fe^2+^/Fe of −0.44 V vs. SHE, Co^2+^/Co of −0.28 V vs. SHE, and Ni^2+^/Ni of −0.25 V vs. SHE) [42, 43] in purifying and Li metal extraction processes, which not only produces selectively the high-purity Li metals from multi-cation electrolytes but can even collect other useful metal products. The critical challenge is finding PEC systems with suitable photoelectrodes to effectively extract various cations in sequence, which is a result of the optimized cathodes at low overpotential to deposit the corresponding metals on the surface and reduce their ion concentration in the electrolytes. Practically, according to a series of investigations, Ni foam cathode and annealing TiO_2_/n^+^p-Si (labeled as ATOS) photocathode are recommended to extract the Fe (Co, Ni) and Li metals with over 1 g h^−1^ m^−2^ of yield rate at low overpotentials in a three-electrode system, respectively. Further integrating with TiO_2_ photoanode, highly efficient extractions of Li and Fe (Co, Ni) metals are achieved at 2- and 0-V applied bias in a solar-driven two-electrode system. A long-term operation and techno-economic assessment (TEA) demonstrates the feasibility of this designed PEC process for metallic Li extraction from waste batteries compared with the conventional and electrochemical process.

## Experimental Section

### Formation of n^+^p-Si with Pyramidal Surface

The preparation of n^+^p-Si with pyramidal surface mainly included an alkali etching process and a phosphorization treatment. First of all, the alkali etching process was carried out. The p-type Si wafer was subjected to ultrasonic cleaning in acetone, ethanol, and deionized water for 10 min, respectively. The cleaned Si wafer was then placed in a 2% potassium hydroxide (KOH) aqueous solution in an 80 °C water bath for 1 h. After etching, the wafer was immediately removed and ultrasonically cleaned in deionized water for 3 min, followed by drying with cold air.

Next, the preparation of the n^+^p junction was performed. The etched Si wafer was immersed in a 5% hydrofluoric acid (HF) solution for 3 min to remove the oxide layer, followed by cleaning with deionized water and drying with cold air. Then, 0.1419 g of phosphorus pentoxide (P_2_O_5_) was dissolved in 10 mL of anhydrous ethanol by ultrasonic treatment. 2 drops of the prepared 0.1 mol L^−1^ P_2_O_5_ solution were applied to the etched surface of the Si wafer (approximately 2 cm^2^), which was then subjected to rapid annealing at 800 °C for 8 min in a vacuum furnace. After the temperature below 300 °C, the sample was removed from the furnace and cleaned in a 5% HF solution for 1 min.

### The Preparation of Si-based Photocathodes

The clean and dry n^+^p-Si with pyramidal surface was placed into the deposition chamber. TiO_2_ thin films were deposited on the n^+^p-Si, clean quartz glass, and FTO wafer using a radio-frequency magnetron sputtering system. The Ti target had a purity of > 99.99 wt%, with a diameter of 60 mm and a thickness of 4 mm. TiO_2_ film on n^+^p-Si (TOS) was deposited by sputtering the target at 150 W RF power in a mixed atmosphere of Ar (99.999%) and O_2_ (99.999%) at a 20 sccm: 20 sccm ratio. The working pressure and deposition time were 2 Pa and 15 min, respectively. A Ti thin film was deposited on the back surface of the n^+^p-Si wafer using Ti target at 200 W DC power for 10 min at a working pressure of 2 Pa.

The W target had a purity of > 99.999 wt%, with a diameter of 60 mm and a thickness of 4 mm. WO_3_ film on n^+^p-Si (WOS) was deposited by sputtering the target at 150 W DC power in a mixed atmosphere of Ar (99.999%) and O_2_ (99.999%) at a 20 sccm: 20 sccm ratio. The working pressure and deposition time were 2 Pa and 15 min, respectively. A Ti thin film was deposited on the back surface of the n^+^p-Si wafer using Ti target at 200 W DC power for 10 min at a working pressure of 2 Pa.

The Mo target had a purity of > 99.999 wt%, with a diameter of 60 mm and a thickness of 4 mm. MoO_3_ film on n^+^p-Si (MOS) was deposited by sputtering the target at 150 W DC power in a mixed atmosphere of Ar (99.999%) and O_2_ (99.999%) at a 20 sccm: 20 sccm ratio. The working pressure and deposition time were 2 Pa and 15 min, respectively. A Ti thin film was deposited on the back surface of the n^+^p-Si wafer using Ti target at 200 W DC power for 10 min at a working pressure of 2 Pa.

The TOS, WOS and MOS photocathodes were further annealed in a rapid annealing furnace at 100 and 200 °C, respectively. The TOS, WOS and MOS photocathodes annealed at 100 and 200 °C were labeled as ATOS and ATOS-200, AWOS and AWOS-200, AMOS and AMOS-200, respectively.

Conductive silver pastes were used to attach a Cu metal strip to the back of the Si wafer, forming an ohmic back contact. After drying, the entire back surface and part of the front surface of the Si-based photocathode were encapsulated with epoxy resin, forming an exposed active area of approximately 0.2 cm^2^. The geometric area of the exposed photoelectrode surface defined by the epoxy resin was determined using calibrated digital images and ImageJ.

The clean and dry FTO wafers were loaded into the deposition chamber. The substrates were cleaned by Ar plasma treatment at 8.6-Pa pressure and 450-V negative bias for 10 min. A metallic Ti target with a diameter of 50.8 mm was used. O_2_ and Ar were introduced into the chamber at a flow rate ratio of 20 sccm: 20 sccm, maintaining a total pressure of 2 Pa. TiO_2_ photoanodes with the thickness of over 1 μm were formed after 5 h of sputter deposition at a DC power of 460 W.

A quartz glass substrate with an area of 3 × 4 cm^2^ was selected, and a 1.5 × 1.5 cm^2^ region at the center of the substrate was covered with silicone gel to prevent the deposition of photoanode materials, which would later be used for mounting the Si-based photocathode. The deposition of a Ti layer was first performed on the quartz glass at a pressure of 2 Pa and a power of 430 W for 5 min, with an Ar flow rate of 40 sccm. Some small solid wafers were used to cover certain contact points to prevent the deposition of other materials, which would be used for the subsequent attachment of a Cu strip. Subsequently, Ar/O_2_ mixed gases were introduced at a ratio of 20 sccm: 20 sccm, and TiO_2_ was deposited using DC power of 460 W for 5 h, maintaining a pressure of 2 Pa during the process. Finally, the solid wafers and silicone gel were removed from the quartz glass, and a Cu strip was attached using silver paste. A 1 × 1 cm^2^ Si-based photocathode was then affixed to the exposed Cu strip in the blank area of the quartz glass. The edges of the photocathode and the exposed Cu strip on the quartz glass were sealed with epoxy resin and dried in air for 2 h, resulting in a coplanar tandem PEC device.

### Physicochemical Characterization

The surface morphology of the thin films was observed using a field emission scanning electron microscope (FESEM). To investigate the crystalline structure of the samples, X-ray diffraction (XRD) measurements were performed using a Bruker D8 Advance instrument with Cu Kα radiation. The 2*θ* range was from 10° to 80°, with a scan step of 1° and a scan rate of 2° min^−1^. The chemical composition of the samples before and after the PEC lithium extraction reaction was analyzed using X-ray photoelectron spectroscopy (XPS) (ESCALAB Xi + , Thermo Fisher Scientific, USA) with monochromatic Al Kα radiation at a pass energy of 29.4 eV. All binding energies were referenced to the C 1*s* peak (284.8 eV) derived from adventitious carbon. To qualitatively determine the presence of Li metal in the samples before and after the reaction, electron paramagnetic resonance (EPR) spectroscopy was applied using x-band (9.2 GHz) and a scanning magnetic field at room temperature. To enable fair comparison of EPR intensities, the mass of all samples was normalized in the EPR data. Inductively coupled plasma mass spectrometry (ICP-MS, Agilent 7900, USA) was employed for the quantitative analysis of Li, Fe, Co, and Ni in the electrolytes after the reaction. For further analysis of the electrolytes before and after the reaction, gas chromatography-mass spectrometry (GC–MS, QP-2020 NX, Shimadzu) was employed using an SH-I-5Sil MS column (length = 30 m, diameter = 0.25 mm, thickness = 0.25 μm, Agilent) at a temperature range of 40–280 °C with a heating rate of 30 °C min^−1^. Optical diffuse reflectance and transmittance were monitored using a UV–Vis–NIR spectrophotometer (Hitachi, UV-4100) at incident angles ranging from 350 to 2600 nm. The absorption coefficient (*α*) of TiO_2_, WO_3_ and MoO_3_ film was derived from the transmission spectra (*T*) and the corresponding thin film thickness (*d*), as shown below:

1 $$\alpha = \frac{{\ln \left( {1/T} \right)}}{\lambda }$$

Optical diffuse reflectance was monitored using an integrating sphere with a vertical incidence wavelength ranging from 250 to 2600 nm on a UV–Visible–NIR spectrophotometer (Hitachi, UV-4100). The Kubelka–Munk theory is generally used for the analysis of diffuse reflection (*R*) spectra to obtain the absorption coefficient (*α*) of the samples on Si wafer as following:

2 $$F\left( R \right) = {\raise0.7ex\hbox{${\left( {1 - R} \right)^{2} }$} \!\mathord{\left/ {\vphantom {{\left( {1 - R} \right)^{2} } {2R}}}\right.\kern-0pt} \!\lower0.7ex\hbox{${2R}$}} \cong \alpha$$

where *F*(*R*) is Kubelka–Munk function. The optical energy bandgap of the sample was calculated by using the classical relation of optical absorption:

3 $$\alpha h\nu = B\left( {h\nu - E_{g} } \right)^{m}$$

where *B*, *Eg* and *hυ* denote as the band tailing parameter, the optical band gap and the photon energy, respectively. The value of *m* should be taken as 0.5 or 2, corresponding to the direct or indirect allowed transition which dominates over the optical absorption, respectively.

### PEC Measurements

PEC measurements were conducted using a three-electrode cell in the PEC 1000 system (PerfectLight Co. Ltd.), where the Si-based photocathode served as the working electrode, a Pt foil as the counter electrode, and an Ag/AgCl electrode as the reference electrode. Solar illumination was provided by a solar simulator (fiber optic source, FX300) with a NIR cut-off filter under AM 1.5G conditions at 100 mW cm^−2^. The range of irradiated wavelengths was from UV to visible light (≤ 800 nm). Prior to each measurement, the solar simulator's intensity was calibrated using a reference silicon solar cell and a solar simulator irradiance meter (PerfectLight Co. Ltd., PL-MW 200). The electrolytes were 0.1 M LiClO_4_-propylene carbonate (PC) solutions. Linear sweep voltammetry (LSV) and cyclic voltammetry (CV) data were collected using a CHI 630E electrochemical workstation without any iR compensation. For typical LSV and CV measurements, the voltage was scanned at rates of 0.01 and 0.05 V s⁻^1^, respectively. In all the PEC processes, Ar gas as protective gas was bubbled in the reaction unit to avoid the oxidation of Li metals. The following relationship was used to convert the Ag/AgCl electrode readings to the reversible hydrogen electrode (RHE).

4 $$E({\mathrm{RHE}}) = E({\mathrm{Ag}}/{\mathrm{AgCl}} ) + 0.197V + 0.059 \times pH$$

The pH value of the electrolyte was measured using specialized pH test strips (pH = 5.4). Additionally, the redox potential of Li^+^/Li relative to the standard hydrogen electrode (SHE) is −3.04 V.

The PEC measurements of the two-electrode device were conducted under 1 sun illumination (100 mW cm^−2^, AM 1.5 G) in a single cell equipped with a flat quartz glass window. The concentration of Li ions in the purified electrolyte was 0.1 M. In the two-electrode system, an ATOS photocathode adhered to a quartz glass substrate and surrounded by a TiO_2_ photoanode was assembled to form a PEC device (ATOS-TiO_2_ device) for Li metal extraction. The sunlight was incident perpendicularly on the side of the device assembly with the photocathode and photoanode, where the ATOS photoanode and TiO_2_ photocathode could simultaneously capture the photons and generate the electrons and holes. The electrons and holes were further separated and migrated to the surfaces of the photoanode and photocathode, respectively, to undergo Li extraction reactions.

In the PEC three-electrode system for Fe, Co, and Ni metal extraction, a TiO_2_ photoanode, Ni foam and Ag/AgCl electrode were used as the counter electrode, working electrode and reference electrode, respectively. PEC measurements were conducted under sunlight illumination (100 mW cm^−2^). In the two-electrode system, the TiO_2_ photoanode and Ni foam still served as the counter electrode and working electrode, respectively. The concentrations of Fe, Co and Ni ions in the electrolyte were 1, 1, and 10 mM, respectively, for corresponding metal extraction. Their current density (*J*)-potential (*V*) curves and *J*-time (*t*) curves were tested on CHI 630E.

To investigate the Li⁺ insertion kinetics and capacity, TiO_2_, WO_3_, and MoO_3_ layers were deposited and annealed on FTO glass using the same deposition and annealing conditions as those for the ATOS, AWOS and AMOS photocathodes, respectively. Cyclic voltammetry (CV) measurements were performed at scan rates of 20, 100, 150, and 200 mV s^−1^ using an electrochemical system to study the kinetics of Li⁺ insertion. The electrolytes were 1 M LiClO_4_-PC solutions. The coloration measurements were recorded using a laser monochromator (Thorlabs LDM670) at a wavelength of 630 nm and an electrochemical workstation (CS350).

The quantitative determination of Li, Co and Ni extraction products was performed using ICP testing. The post-electrolysis electrolytes of 100 μL were took and diluted with 5% nitric acid of 9.9 mL before the ICP measurements. Based on the test results and the known concentrations of metallic ions, the standard curves were plotted. The quantification of Fe ions was conducted directly by measuring the absorbance of the electrolyte using a UV–Vis spectrophotometer. The absorbance of the UV–Vis spectrophotometer at a wavelength of 362 nm was linearly related to the concentration of Fe ions. The standard curves of Fe ions were plotted. The extraction yield and Faradaic efficiency of PEC extraction were calculated by the following equations:

5 $${\mathrm{Extraction}}\;{\mathrm{rate}}\left( {g \cdot h^{{ - 1}} \cdot m^{{ - 2}} } \right) = \frac{{C_{{\mathrm{M}}} \times V}}{t \times A}$$

6 $${\mathrm{FE}} = \frac{{C_{{\mathrm{M}}} \times V \times n \times F}}{M \times Q} \times 100\%$$

In the equations, *C*_M_, *V*, *t*, *A*, *n*, *F*, *M* and *Q* represent the concentration of metallic ions in the electrolyte, the volume of the reaction electrolyte, the time for metal extraction, the surface area of photocathode or cathode, the number of electrons transferred, the Faraday constant (96,500 C mol^−1^), the relative molecular mass of the product, and the total electrical charge applied.

## Results and Discussion

### Screening Si-based Photocathode for PEC Li Metal Extraction

As reported in electrochromic field, TiO_2_, WO_3_ and MoO_3_ are known as promising candidates for facilitating the insertion of Li ions into their lattices and activating the Li ions at the surface under applied negative bias [44–46]. In this work, we chosen TiO_2_, WO_3_, and MoO_3_ as the cocatalyst materials to systemically investigate the behaviors of PEC Li metal extraction and corresponding work mechanism. A planar p-type Si wafer underwent an alkaline etching and a phosphorization treatment to obtain the n^+^p-Si light absorber with pyramidal surface, as usually served in photovoltaic industry [47]. A cocatalyst layer was deposited on the n^+^p-Si surface and annealed at 100 °C. The annealing TiO_2_/n^+^p-Si, WO_3_/n^+^p-Si and MoO_3_/n^+^p-Si photocathodes are simply labeled as ATOS, AWOS and AMOS, respectively. Detailed preparation processes of these samples and other samples with different preparation conditions are described in Fig. S1. FESEM images with EDS mapping (Figs. S2-S4) and XRD patterns (Fig. S5) of the photocathodes reveal that the cocatalyst layer with nanograins is conformal by pyramidal geometry coating on the n^+^p-Si. Successful loading of the TiO_2_, WO_3_ and MoO_3_ layer onto Si-based photocathodes was verified by XPS (Fig. S6) with the presence of Ti^4+^, W^6+^ and Mo^6+^, respectively [41, 48, 49]. In the optical measurements (Fig. S7), the photocathodes and their cocatalyst layers showed similar optical properties with low reflectance (~ 10%) and high transmittance (> 80%). According to indirect allowed transition, the band gap of n^+^p-Si, TiO_2_, WO_3_, and MoO_3_ are 1.12, 3.55, 3.00, and 3.16 eV, respectively, in line with the previous reports [50, 51].

**Fig. 2:**
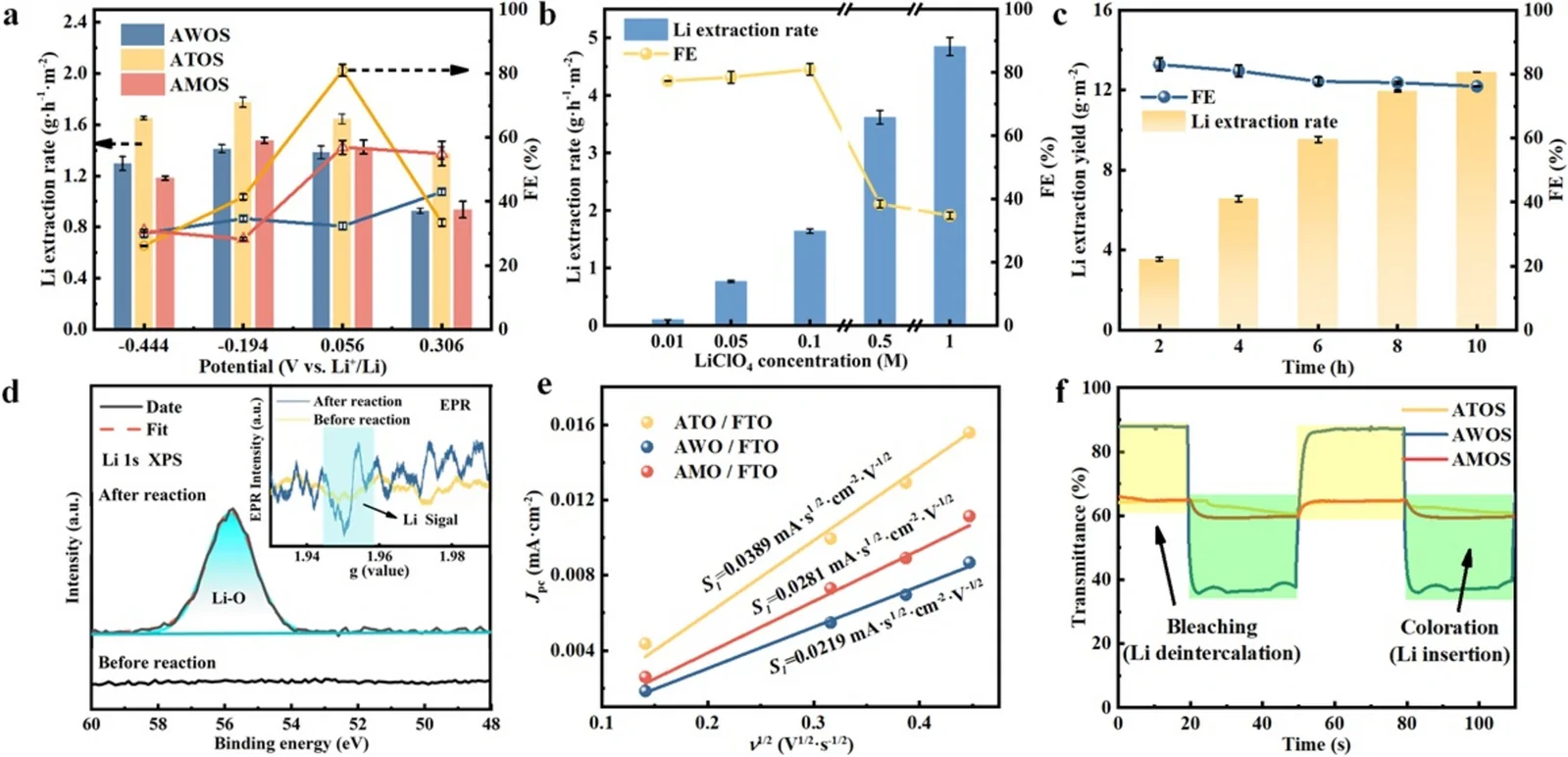
PEC performance of Li metal extraction on the Si-based photocathodes. **a** Li extraction rate (column chart) and FE (scatter plot) on the AWOS, ATOS and AMOS photocathode in 0.1 M LiClO_4_-PC solution at given potentials. **b** Li extraction rate (column chart) and FE (scatter plot) on the ATOS photocathode in the solution with different concentrations of LiClO_4_ at 0.056 V vs. Li^+^/Li. **c** Time dependence of Li extraction rate (column chart) and FE (scatter plot) obtained from ATOS photocathode in 0.1 M LiClO_4_-PC solution at 0.056 V vs. Li^+^/Li. These measurements of PEC Li metal extraction were carried out in the LiClO_4_-PC solution in a three-electrode system for 4 h under 1 sun illumination. **d** Li 1*s* XPS spectra of the ATOS photocathode before and after PEC Li metal extraction. The inset is EPR spectra of the ATOS photocathode before and after PEC reaction. **e** Plots of peak current density (*J*_pc_) vs. square root of the scanning rate recorded in the AWO, ATO and AMO layer on FTO substrates. **f** Coloration/bleaching switching response curves of the AWO, ATO and AMO layer detected at the wavelength of 630 nm

To quickly evaluate the Si-based photocathodes, the measurements of PEC Li metal extraction in this section were only carried out in 0.1 M LiClO_4_-PC solution in a three-electrode system for 4 h under 1 sun illumination, which have been developed in our prior study [41] with slight modifications including the reaction cell (Fig. S8), calibration curve via ICP-MS (Fig. S9), CV analysis (Fig. S10), chopped *J*-*V* curves (Fig. S11), and *J*-*t* curves (Fig. S12). Before comparing the performance of the PEC Li metal extraction on three types of Si-based photocathodes, each type of photocathode with different deposition times and annealing temperatures were explored to optimize its PEC behaviors. As shown in Figs. S13-S16, the optimal deposition time and annealing temperature were 15 min and 100 °C to be used in the preparation process of ATOS, AWOS, and AMOS. During the extraction process, some fine Li metal nanoparticles were precipitated on the photocathode surface and gradually and continuously stacked to form the big particles with the increase in reaction time (Fig. S17). After 4 h of PEC reaction, the photocathode with these Li metal particles intensely reacted with H_2_O and made a clear voice with glaring spark (Video S1). The Li extraction rate and FE of ATOS, AWOS, and AMOS photocathodes at certain potential range in a 4-h period are shown in Fig. 2a. The Li extraction rate and FE were in the range of 1.38–1.77 g h^−1^ m^−2^ and 26.2%–81.0% on the ATOS photocathode, respectively, which are higher than those on the AWOS (0.92–1.41 g h^−1^ m^−2^ and 29.9%–43.0%) and AMOS (0.93–1.47 g h^−1^ m^−2^ and 28.2%–56.9%) photocathodes. When the applied potential shifted toward to 0.056 V vs. Li^+^/Li, the ATOS photocathode showed an optimum performance of PEC Li metal extraction with an acceptable extraction rate (1.64 g h^−1^ m^−2^) and a maximum FE (81.0%). Beyond this applied potential, the FE decreased markedly, resulting from the competitive reaction on the photocathode surface (i.e., self-reduction of the photocathode). Thus, the ATOS sample is employed as the prototype photocathode to study the follow-up Li metal extraction. As discussing in previous Li ion extraction work [8], the effect of Li concentration on PEC Li metal extraction needs to be investigated to define the application range of Li recovery by the sustainable method. Figures 2b and S18 reveal the behaviors of PEC Li metal extraction in the electrolytes with different Li concentrations. The Li extraction rate increased sharply from 0.1 to 1.64 g h^−1^ m^−2^ with the increase of Li concentration from 0.01 to 0.1 M, and its increment gradually slowed down as the Li concentration exceeded 0.1 M. The Li extraction rate can reach at 4.85 g h^−1^ m^−2^ on the ATOS photocathode in the 1 M LiClO_4_-PC electrolytes. In addition, a high FE (~ 80%) was kept in the low Li concentration (≤ 0.1 M), and an obvious reduction of FE (~ 40%) in the high Li concentration can be attributed to the presence of competing reaction. The stable operation of the Si-based photocathodes is a key factor to be considered in the application of PEC Li metal extraction. As shown in Fig. S19, the *J*-*t* curve of ATOS photocathode was relatively constant in the 6-h PEC extraction process, and a slight decrease of *J* occurred in the longer operation time due to the accumulation of Li metals for obstructing the light transmission. The metallic Li production as a function of reaction time at 0.056 V vs. Li^+^/Li is shown in Fig. 2c. It is clearly found that the Li extraction yield with almost stable FE (> 76%) was increased progressively with the increase in reaction time, which reached 12.9 g m^−2^ in a 10-h period.

After extraction reaction, XPS was used to analyze the chemical composition and electron structure of the photocathode surface. The high-resolution Li 1*s* spectra (Fig. 2d) reveal that there was no XPS peak on the ATOS photocathode before reaction, meaning the absence of Li. Different from the fresh photocathode, ATOS photocathode after extraction reaction showed a strong XPS signal of Li at 55.8 eV, demonstrating the presence of Li_2_O [41]. The formation of Li_2_O on the photocathode surface is ascribed to the Li metals exposing in the air. The full survey scan and O 1*s* spectrum of ATOS photocathode after reaction provided the corresponding information on the Li extraction and the disappearance of Ti and Si peaks (Fig. S20). Similar phenomenon was also observed in the XPS spectra of the reacted AWOS and AMOS photocathode (Figs. S21 and S22). These XPS results accord with a thick Li metal layer coating on the photocathode surface. To further determine the formation of Li metal layer qualitatively, EPR spectroscopy as a nondamaged and sensitive technique was used to detect the state of the photocathode with unpaired electrons. As recorded in the inset of Fig. 2d, a visible EPR peak, due to the presence of Li metal layer with free electrons, was exhibited in the reacted ATOS photocathode [52]. Similar signal was also found in other Si-based photocathodes after PEC Li metal extraction (Fig. S23). The kinetics and capacity of Li insertion for various cocatalyst layers on FTO glasses were studied by electrochemical CV behaviors at different scan rates (Figs. 2e and S24) and coloration measurements (Fig. 2f), respectively, to understand the catalytic mechanism for Li metal extraction. According to the relationship of the scan rates vs. the peak current, the diffusion coefficient of Li ions in the cocatalyst layer can be calculated by the Randles–Sevcik equation [53]:

7 $$J_{{{\mathrm{pc}}}} = 2.69 \times 10^{5} n^{{{\raise0.7ex\hbox{$3$} \!\mathord{\left/ {\vphantom {3 2}}\right.\kern-0pt} \!\lower0.7ex\hbox{$2$}}}} D^{{{\raise0.7ex\hbox{$1$} \!\mathord{\left/ {\vphantom {1 2}}\right.\kern-0pt} \!\lower0.7ex\hbox{$2$}}}} \nu^{{{\raise0.7ex\hbox{$1$} \!\mathord{\left/ {\vphantom {1 2}}\right.\kern-0pt} \!\lower0.7ex\hbox{$2$}}}} C{}_{0}$$

where *J*_pc_, *n*, *D*, *ν* and *C*_0_ are the peak current density, number of transferred charges, diffusion coefficient of Li ions, potential scan rate and concentration of Li ions, respectively. As shown in Fig. 2e, a linear relationship between the peak current density and the square root of scan rate is built from the diffusion-control intercalation processes of Li ions in all cocatalyst layers. In comparison with AWO and AMO layer, ATO layer showed a higher diffusion coefficient of Li ions (Table S1), reflecting that the lattice structure of TiO_2_ has better electrochemical kinetics of the Li ion insertion. Namely, TiO_2_ as cocatalyst layer can be a benefit for the enrichment and activation of Li ions on the surface to promote the reduction of Li ions by accepting the photogenerated electrons. Meanwhile, during the coloration measurements, the transparent state of oxide layer is bound up with the number of Li ion insertion [45]. Figure 2f demonstrates that the ATO layer allows less Li ions intercalating into the lattices than AWO and AMO layer, implying that the inserted Li ions are concentrating on the ATO surface. As discussed in our previous work [41], the intercalated Li ions on the surface can fast attend in the reduction reactions to produce the Li metals. In addition, a reversible intercalation/de-intercalation process was found in these cocatalyst layers, meaning that the Si-based photocathodes can be refreshed by applying a positive potential and repeatedly used in the PEC Li metal extraction. In a word, TiO_2_ layer can be considered as a promising cocatalyst layer on the Si-based photocathode for efficient PEC Li metal extraction.

### Design and Demonstration of Selective Li Metal Extraction

**Fig. 3:**
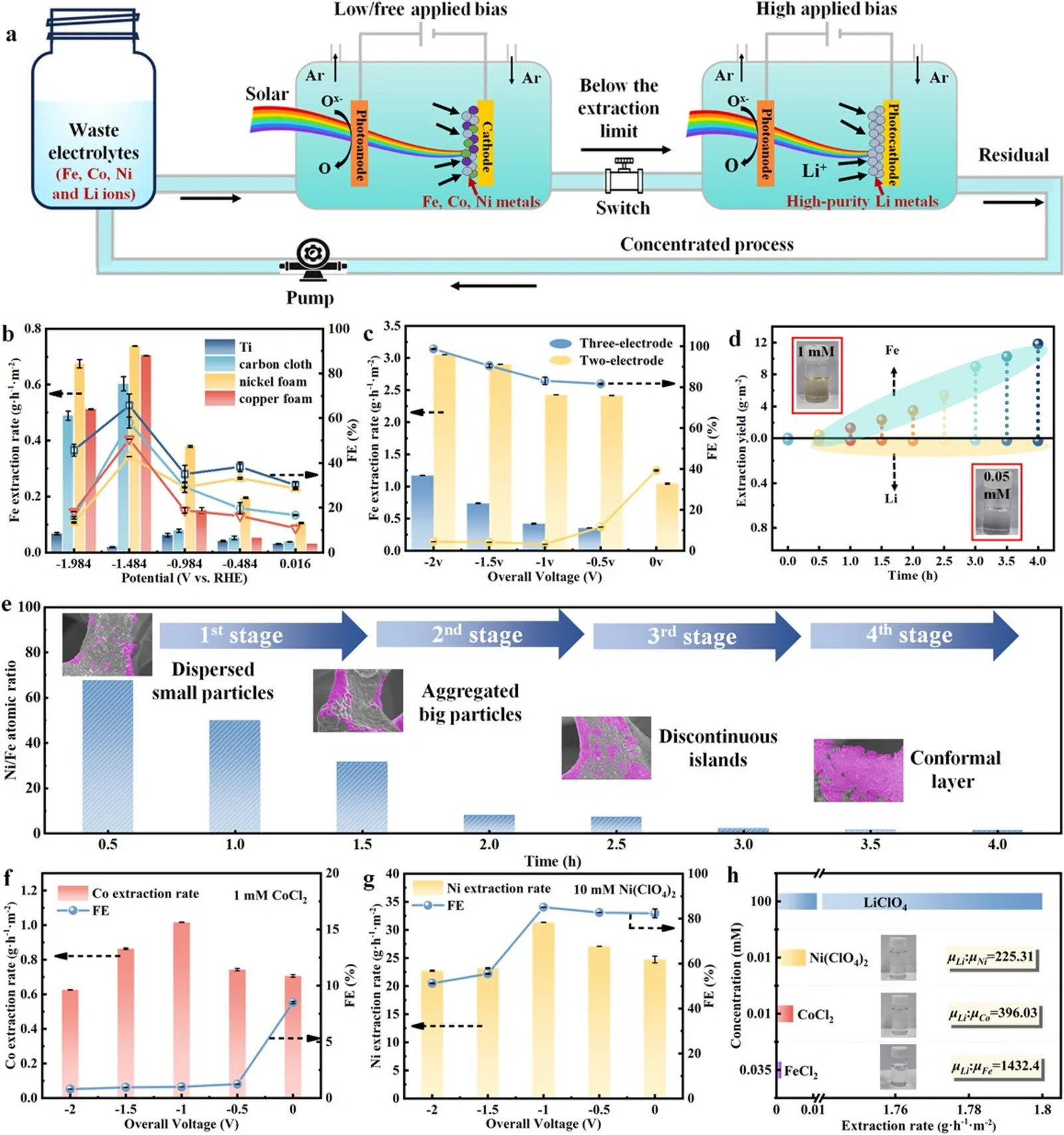
Process design for selective Li extraction from the multi-cation electrolytes. **a** Design of the selective Li extraction from multi-cation electrolytes by PEC method. **b** Fe extraction rate (column chart) and FE (scatter plot) on the Ti plate, carbon cloth, Ni foam and Cu foam as the cathodes in 1 mM FeCl_2_-PC solution by electrochemical method. These electrochemical measurements were carried out in 1 mM FeCl_2_-PC solution in a three-electrode system for 2 h at given potentials. In the system, the counter electrode was Pt mesh, as described in the Methods of SI. **c** Performance comparison of PEC Fe metal extraction in three- (blue) and two-electrode (orange) systems with Ni foam cathode and TiO_2_ photoanode in 1 mM FeCl_2_-PC solution for 2 h. **d** Time dependence of PEC Fe and Li extraction yield obtained from bias-free Ni foam-TiO_2_ photoanode system in 0.1 M LiClO_4_-1 mM FeCl_2_-PC solution. The upper left and lower right insets are the graphs of the electrolytes after 0-h and 4-h reactions including 1-mM and 0.05-mM Fe ions, respectively. **e** Ni/Fe atomic ratio (column chart) and corresponding FESEM images of the Ni foam cathode after bias-free PEC extraction with different reaction times. **f** Co extraction rate (column chart) and FE (scatter plot) in a Ni foam-TiO_2_ photoanode two-electrode system in 1 mM CoCl_2_-PC solution at given potentials for 4 h. **g** Ni extraction rate (column chart) and FE (scatter plot) in a Ni foam-TiO_2_ photoanode two-electrode system in 10 mM Ni(ClO_4_)_2_-PC solution at given potentials for 4 h. **h** Extraction rate of various metals from double-cation electrolytes with 0.1 M LiClO_4_ by PEC methods with the ATOS photocathode at 0.056 V vs. Li^+^/Li as the concentrations of Fe, Co and Ni ions reach the extraction limit. The insets are the corresponding photographs of double-cation electrolytes with Li-Ni ions, Li-Co ions and Li-Fe ions from top to bottom. All the PEC measurements were carried out under 1 sun illumination

In addition to the valuable Li ions, there are many other metal ions (i.e., Fe, Co, and Ni) in the real waste Li-containing batteries. It can be imagined that the presence of the foreign metal ions in the electrolytes disturbs the PEC Li metal extraction process and reduces the purity of metallic Li production. Therefore, selectively extracting Li metals from the multi-cation electrolytes is a necessary and sufficient condition for developing large-scale and practical PEC Li recovery technology. As mentioned in the Introduction section, the different metallic elements usually possess different redox potentials (i.e., Li^+^/Li of −3.04 V vs. SHE, Fe^2+^/Fe of −0.44 V vs. SHE, Co^2+^/Co of −0.28 V vs. SHE, and Ni^2+^/Ni of −0.25 V vs. SHE), and such difference of redox potential between Li and other metallic elements can provide an opportunity for separately obtaining high-purity Li metal and other metal production. Considering the ubiquity and abundance of Fe, Co and Ni in the waste electrolytes, these ions were chosen as the impure cations in the electrolytes to investigate the selectively PEC extracting behaviors of Li metals. The working principle and process of selective Li metal extraction are illustrated schematically in Fig. 3a. The feeding electrolytes are firstly purified through an applicable PEC system extracting the impure cations at zero or low applied bias. When the concentration of impure cations is too low enough to be availably extracted and influences the purity of Li metals in the conditions of PEC Li metal extraction (simply defined as extraction limit), the purified electrolytes flow into the second reaction cell to implement the efficient PEC Li metal extraction, as achieved in the previous section. The residual electrolytes after Li metal extraction are concentrated and pumped into the feeding electrolytes to attend in the next extraction cycle (Fig. 3a). Currently, to realize the selective Li metal extraction in this plan, two core issues need to be addressed: (1) Exploiting cheap materials as cathode to absorb the impure cations and obtain the corresponding metal production at low overpotentials; (2) Assembling with the photoelectrode to conduct a bias-free PEC extraction behavior under solar irradiance.

To quickly screen a suitable cathode for Fe metal extraction, we first carried out a series of electrochemical Fe metal extraction measurements on commercial titanium (Ti) plate, carbon cloth, Ni foam and Cu foam in 1 mM FeCl_2_-PC solution. The standard solutions of Fe, Co and Ni ions with known concentrations were prepared, and their calibration curves were obtained by UV–visible–near-IR spectrophotometer and ICP-MS (Fig. S25), respectively. Figure 3b shows the typical performance of Fe metal extraction on various cathodes as a function of applied potential in a three-electrode system for 2 h. An ignorable Fe extraction rate (less than 0.07 g h^−1^ m^−2^) with a considerable FE was observed on the Ti plate in the potential range of −1.984‒0.016 V vs. RHE. Compared with the carbon cloth (0.04‒0.60 g h^−1^ m^−2^ of extraction rate) and Cu foam (0.03‒0.70 g h^−1^ m^−2^ of extraction rate), Ni foam displayed a better Fe extraction performance with higher extraction rate (0.11‒0.74 g h^−1^ m^−2^) and similar FE. As a result, the Ni foam will be used as cathode to extract the Fe, Co and Ni metals by PEC method with TiO_2_ photoanode in three- and two-electrode systems. The experimental setup for PEC extraction of Fe, Co and Ni metals is shown in Fig. S26. As reported in our previous work [50, 54], the simple and cheap TiO_2_ photoanode presented a saturated photocurrent density of 0.5 mA cm^−2^ at 0.3 V vs. RHE and an early onset potential of −0.1 V vs. RHE for oxygen evolution reaction in a 1 M KOH solutions (Fig. S27a). Figures S27b-c and S28 show PEC behaviors of Fe metal extraction in three- and two-electrode systems with Ni foam cathode and TiO_2_ photoanode in 1 mM FeCl_2_-PC solution, respectively. Due to the lack of reference electrode in two-electrode system, the directly applied potential (namely overall voltage) is used to displace the Li^+^/Li or RHE and discussed in the remaining part. It is easily found in Fig. 3c that PEC Fe metal extraction in two-electrode system had higher extraction rate (1.04‒3.05 g h^−1^ m^−2^) and lower FE (less than 40%) than that in three-electrode system (0.36‒1.17 g h^−1^ m^−2^, more than 80%). This can be attributed higher photocurrent density in two-electrode system. Considering comprehensively the solar energy utilization and extraction efficiency (1.04 g h^−1^ m^−2^ and 39%), bias-free PEC two-electrode system with Ni foam cathode and TiO_2_ photoanode is recommended to extract the impure cations. In this situation, we further studied the time dependence of PEC Fe and Li extraction yield by the bias-free system in 0.1 M LiClO_4_-1 mM FeCl_2_-PC solution (Fig. 3d). There was a nearly linear increase in the extraction yield of Fe metals in a 4-h PEC period, implying that the Fe metal extraction stably operates. A high extraction yield of 11.8 g m^−2^ was obtained on the two-electrode system at 4 h, and the concentration of Fe ions after 4 h reduced to about 0.05 mM corresponding to the colorless electrolytes (the lower right inset of Fig. 3d) and the reacted Ni foam with brownish red coating (Fig. S29). The brownish red coating on the Ni foam can be derived from the extracted Fe metals oxidized in the air. As expected in our plan, the extraction yield of Li metal in this bias-free PEC system is almost zero (see Fig. 3d), meaning that a zero-loss Li resource occurs in the Fe extraction process. To make clear the evolution of composition and structure in the extraction process, FESEM with EDS mapping was used to analyze the surface of Ni foam after PEC reactions with different times (Figs. 3e, S30 and S31). With the increase in reaction time from 0.5 to 4 h, the atomic ratio of Ni and Fe significantly reduced from 67.6 to 1.4, demonstrating the increase of Fe metal deposits. The deposition process of Fe metals on the Ni foam can be grouped into four stages: small particles dispersed on the surface, the aggregation of small particles to big particles, the formation of discontinuous islands, and the presence of conformal Fe layer. Actually, these stages in the Fe metal extraction process are in agreement with the preparation of metallic films by electrodeposition [55] and can be supposed to appear in the extraction process of Ni and Co metals.

If the other impure cations can also be efficiently extracted in the current bias-free system, selective PEC Li metal extraction will be achieved in the multi-cation electrolytes. PEC extraction of Co and Ni metals was measured in the Ni foam-TiO_2_ photoanode two-electrode system for 4 h in 1 mM CoCl_2_-PC (Fig. S32) and 10 mM Ni(ClO_4_)_2_-PC solutions (Fig. S33), respectively. Although the maximum extraction rate of Co metal (1.02 g h^−1^ m^−2^) was found in the PEC two-electrode system at −1.0 V, the optimal performance of Co metal extraction with an acceptable extraction rate (0.71 g h^−1^ m^−2^) and the highest FE (8.5%) still occurred at zero applied potential (see Fig. 3f). The color of electrolytes was from blue to colorless matching with the color of Ni foam from gray to black (the color of Co metals oxidized in the air) when the reaction time increased from 0 to 4 h in the bias-free PEC system (Fig. S34). In addition, the relatively low FE and its variation trend with applied potential in the PEC Co metal extraction are similar to those in the Fe metal extraction, which could be attributed to low concentration of Fe and Co ions (1 mM) in the electrolytes. To verify the influence of metal ions on FE, 10 mM Ni(ClO_4_)_2_-PC solutions as the electrolytes were served in the PEC Ni metal extraction. As shown in Fig. 3g, the FE values in the PEC Ni metal extraction were over 50% at all the applied potentials, and the corresponding extraction rates also increased to 22–32 g h^−1^ m^−2^. Importantly, the PEC Ni metal extraction at zero applied potential exhibited 24.7 g h^−1^ m^−2^ of extraction rate and 82.4% of FE. Similar changes on the color of electrolytes and Ni foam were also observed before and after the PEC Ni metal extraction (Fig. S35). Consequently, highly efficient extraction of Fe, Co and Ni metals in the bias-free PEC system can take the first step toward selective Li metal extraction from the multi-cation electrolytes. Besides, a defined extraction limit for each impure cation in the conditions of PEC Li metal extraction is very important and indispensable to determine the purity of Li metal production obtained from the multi-cation electrolytes. Figure 3h is the extraction rate of various metals from double-cation electrolytes with 0.1 M LiClO_4_ by PEC method on the ATOS photocathode at 0.056 V vs. Li^+^/Li as the concentrations of Fe, Co, and Ni ions are 0.035, 0.01, and 0.01 mM, respectively. In the optimal conditions of PEC Li metal extraction, the extraction rates of Li, Fe, Co, and Ni metals on the ATOS photocathodes were 1.80, 0.001, 0.004 and 0.008 g h^−1^ m^−2^, respectively. According to the calculations, the extraction ratio of Li/Fe, Li/Co and Li/Ni is ~ 1432, ~ 396, and ~ 225, respectively, demonstrating that the purity of the obtained Li metal production is over 99.5% in this situation. Therefore, 0.035, 0.01, and 0.01 mM of Fe, Co and Ni ion concentrations are defined as their extraction limits using in the next section to purify the impure cations in the multi-cation electrolytes for PEC extraction of high-purity Li metals.

**Fig. 4:**
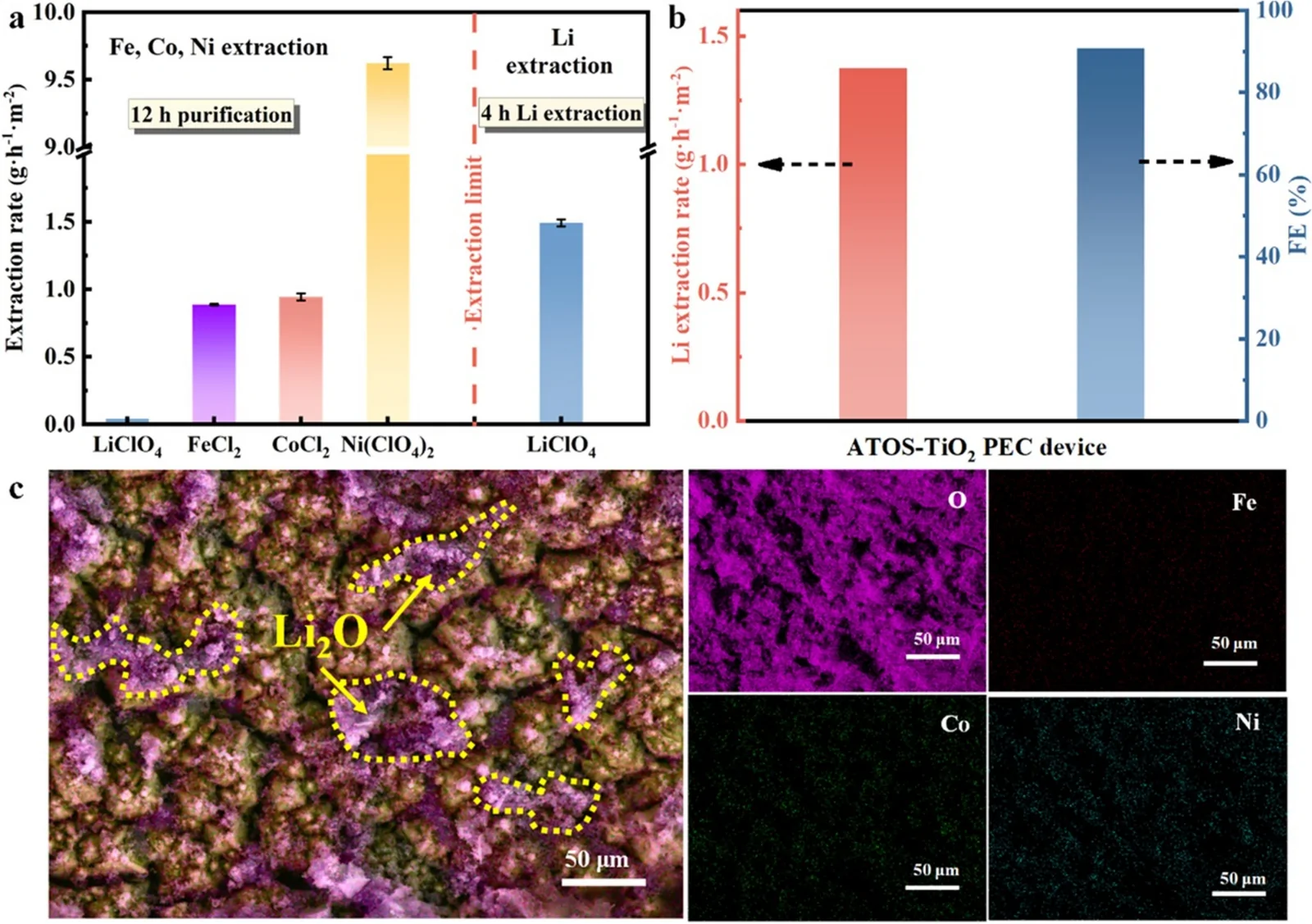
Demonstration of selective Li metal extraction from the multi-cation electrolytes via two ultralow-potential PEC systems. **a** Extraction rates of various metals from the multi-cation electrolytes with 0.1 M LiClO_4_, 1 mM FeCl_2_, 1 mM CoCl_2_ and 10 mM Ni(ClO_4_)_2_ including a purifying process and Li metal extraction process. The purifying process was carried out in PEC two-electrode system with Ni foam cathode and TiO_2_ photoanode at zero applied potential for 12 h under 1 sun illumination. The Li metal extraction process was carried out in PEC three-electrode system with ATOS photocathode at 0.056 V vs. Li^+^/Li for 4 h under 1 sun illumination. **b** Li metal extraction rate and FE on a ATSO photocathode-TiO_2_ photoanode device in 0.1 M LiClO_4_-PC electrolytes at -2.0 V of applied potential for 4 h under 1 sun illumination. **c** Top-view FESEM image and corresponding EDS mapping of ATOS photocathode surface after the Li metal extraction process from the purified multi-cation electrolytes

On basis of the proposed design (Fig. 3a), we investigated the extraction behaviors of high-purity Li metals from the multi-cation electrolytes with 0.1 M LiClO_4_, 1 mM FeCl_2_, 1 mM CoCl_2_ and 10 mM Ni(ClO_4_)_2_ including a purifying process and Li metal extraction. The purifying process was implemented in the bias-free PEC two-electrode system with Ni foam cathode and TiO_2_ photoanode under 1 sun illumination (Fig. S36a). In the 12-h purifying process, notable extraction rates of Fe, Co, and Ni metals were 0.89, 0.93, and 9.65 g h^−1^ m^−2^, respectively, while the deposition of Li metals was negligible (see the left of Fig. 4a). After the purifying process, the concentrations of Fe, Co and Ni ions were reduced to 0.038, 0.025, and 0.017 mM, respectively, which are close to their extraction limit. Visually, the color of electrolytes varied from bluish yellow to colorless as the reaction time was from 0 to 12 h (the insets of Fig. S36a). Subsequently, the Li metal extraction process was quickly carried out in the residual electrolytes by using PEC three-electrode system with ATOS photocathode at 0.056 V vs. Li^+^/Li (being equal to −3.5 V of applied potential) for 4 h (Fig. S36b). The extraction rate of Li metals on the ATOS photocathode in such multi-cation electrolytes still kept 1.50 g h^−1^ m^−2^, implying a good replicability on PEC Li metal extraction. To further reduce the addition of applied bias, a wired PEC device was assembled by attaching the ATOS photocathode to the quartz glass and surrounding with TiO_2_ photoanode (ATOS-TiO_2_, see Fig. S37a). As reported in our previous work [51], the coplanar photocathode and photoanode in the tandem device can decrease the interfacial resistance and accelerate the mass and charge transfer. Under 1 sun illumination, the coplanar ATOS-TiO_2_ device exhibited an excellent light response, a good stability and a high photocurrent (approximate 1 mA) at −2.0 V of applied potential (as shown in Fig. S37). In a 4-h PEC Li metal extraction process, the ATOS-TiO_2_ device achieved a decent extraction rate of Li metals (~ 1.38 g h^−1^ m^−2^) and an outstanding FE (90.7%) at −2.0 V of applied potential (Fig. 4b). Therefore, incorporating the ATOS-TiO_2_ device for the Li metal extraction with the bias-free two-electrode system for the purifying process, selectively producing the high-purity Li metals can be realized in the ultralow-potential combined processes from the waste batteries with various impure cations. In addition, to exclude the presence of impure metals on the surface of ATOS photocathode, FESEM with EDS mapping was employed to investigate the composition and structure of ATOS photocathode surface after the Li metal extraction process from the purified multi-cation electrolytes. It is clearly observed in Fig. S38 that ATOS photocathode still remained the pyramidal surface after 4-h PEC operation, reflecting a low loss of the photocathode in the Li metal extraction process. Many discontinuous islands were loaded on the pyramidal surface. According to the EDS mappings of Fe, Co and Ni elements (see Fig. 4c), there was no corresponding metal on the ATOS photocathode surface. Considering the difference between the mapping of O element and the pyramidal surface with TiO_2_ layer, we can judge the presence of Li_2_O islands, originating from the oxidation of Li metals exposing in the air. In summary, an efficient and selective Li metal extraction from the multi-cation electrolytes can be realized by a combined PEC process with ultralow applied potential.

### Long-Term Stability Test and TEA

**Fig. 5:**
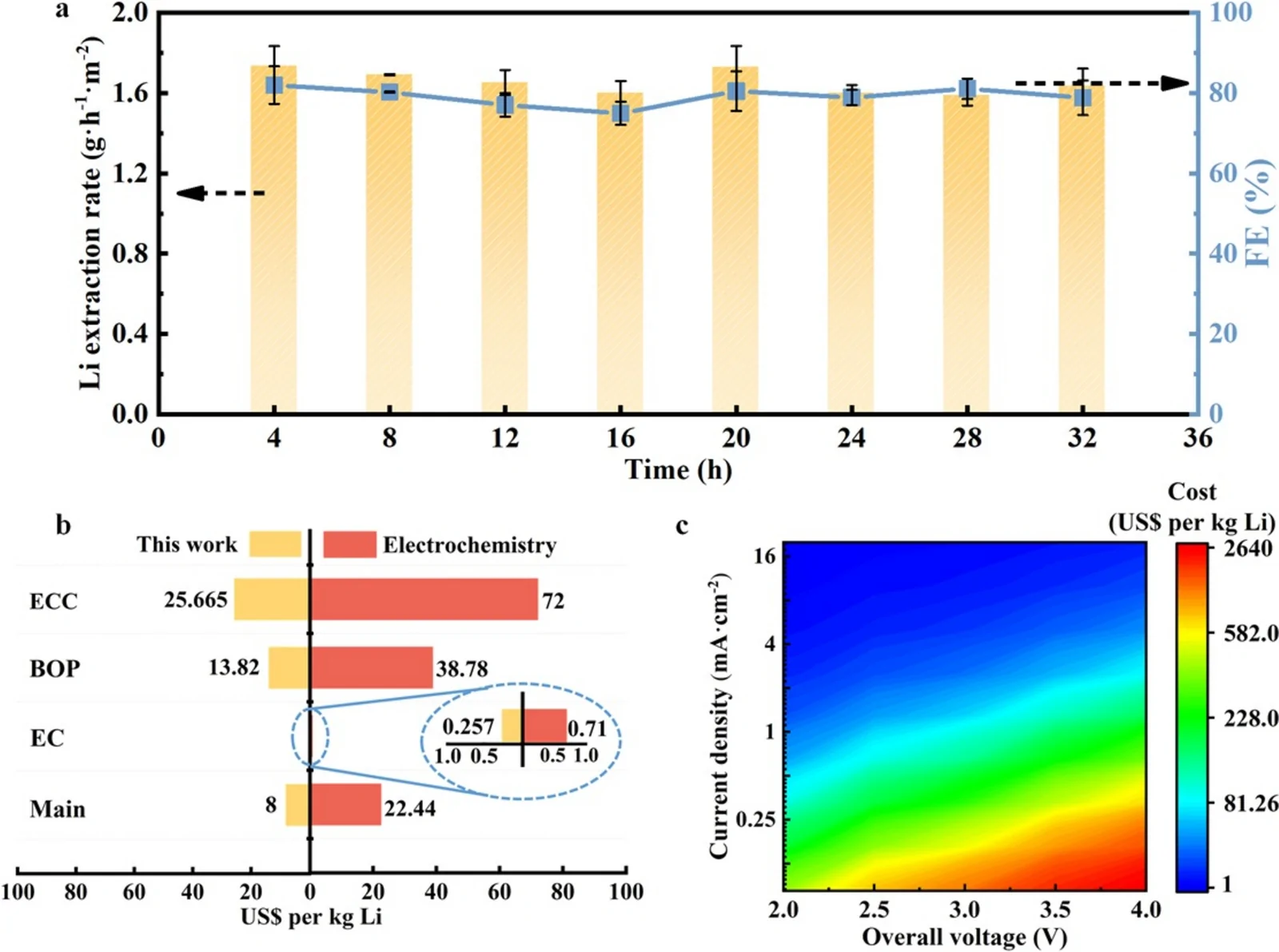
Long-term stability test and techno-economic assessment for PEC Li metal extraction. **a** Extraction rate (column chart) and FE (scatter plot) of Li metals on the ATOS photocathode from the purified electrolytes for eight continuous cycles, where each cycle operates for 4 h. After each cycle, the different batches of ATOS photocathodes were replaced in turn. In the whole testing process, the purified electrolytes with 0.1 M LiClO_4_ were added in the reaction cell. PEC measurements were carried out in a three-electrode system at 0.056 V vs. Li^+^/Li under 1 sun illumination. **b** Extraction cost changes of Li metals by our work and electrochemical method for various parameters with a single-variable sensitivity analysis. The baseline parameters including ECC (electrolytic cell cost, US$ per kg Li), BOP (balance of plant cost, US$ per kg Li), EC (electricity cost, US$ per kg Li) and Main (maintenance cost, US$ per kg Li) were chosen on the basis of the performances demonstrated in this study (Table S2 and S3), and the optimistic and pessimistic parameters were shown as the values next to the orange and red bars, respectively. **c** A contour map for the total costs of Li metal production by our work and electrochemical method with respect to the different current densities and overall voltages. All other parameters are fixed as the baseline parameters in (**b**)

After optimizing all the reaction conditions and the operation processes above, we conducted a cycle operation measurement to validate the practical Li metal extraction of our PEC system design. In the whole selective Li extraction process from the multi-cation electrolytes (Fig. 3a), the Si-based photocathodes can be the most important limiting factor due to the photo-/electro-corrosion of Si and the failure of cocatalyst layer (i.e., lattice expansion by reversible intercalation of Li ions), which usually show shorter service life [56] than the commercial Ni foams and TiO_2_ photoanodes (over 1000 h) [50]. Thus, after each cycle, the different batches of ATOS photocathodes were replaced in turn to evaluate the stability and repeatability of preparation technology, and the purified electrolytes with 30 mL of 0.1 M LiClO_4_ were used in the whole testing process, as depicted in Fig. 5a. During the semi-continuous operation of 8 cycles of PEC Li metal extraction, the extraction rate and FE on the different ATOS photocathodes only showed a slight fluctuation and were maintained in the range of 1.59‒1.73 g h^−1^ m^−2^ and 75%‒82%, respectively (Fig. 5a). The average extraction rate and FE on the ATOS photocathode were 1.65 g h^−1^ m^−2^ and 79.2% throughout the 8 cycles, respectively. The PEC performance of these ATOS photocathodes also remained stable, including similar onset potentials, saturated photocurrent densities and *J*-*t* curves in the cycle measurements (Fig. S9). Please note that the potentiostatic operation here was set at 0.056 V vs. Li^+^/Li under 1 sun illumination on the basis of the guidance above (Fig. 2b) by estimating the optimal applied potentials at different Li metal extraction levels. Overall, from a perspective of sustainable technology, the designed PEC reaction system with purification and Li metal extraction steps can successfully provide a solar-driven and ultralow-potential green method for efficiently and selectively recycling the Li resources from the waste batteries.

With the promising extraction performance of the demonstrated PEC reaction system, a TEA was further performed to appraise the feasibility of the system and whether this sustainable system is worth development and commercialization (Note S1). Note that, in this preliminary model, the original data of electrochemical Li metal extraction reported by Yang et al. [40] were selected as the comparative object to discuss the cost of our work and electrochemistry. A single-variable sensitivity analysis in this work and electrochemical method was implemented to deeply understand the effect of main parameters on the cost (as shown in Figs. 5b and S40). The baseline parameters including ECC (electrolytic cell cost, US$ per kg Li), BOP (balance of plant cost, US$ per kg Li), EC (electricity cost, US$ per kg Li) and Main (maintenance cost, US$ per kg Li) were chosen on the basis of the performances demonstrated in this study (Tables S2 and S3). The sensitivity analysis results indicate that the cost of Li metal extraction in our work and electrochemical method is mainly influenced by ECC, BOP, and Main (Fig. 5b), which are closely related to the cost of electrode materials. In comparison with the electrochemical method, the designed PEC Li metal extraction system showed a lower cost due to the substitution of expensive noble metal electrode (i.e., iridium electrode) by TiO_2_ photoanode and ultralow applied potential (2 V, see Fig. 4b). This result highlights the advantages of PEC system for Li metal extraction to recycle the Li resources and the direction for future studies to reduce the cost. Furthermore, a clear target is clearly observed by plotting the relationship between the cost and extraction performance in a contour map (Fig. 5c). For example, if a better performance of PEC Li metal extraction with cheap photoelectrodes (i.e., *α*-Fe_2_O_3_ photoanode and Cu_2_O photocathode) can be realized under 1 sun illumination in future, such as 2 mA cm^−2^ at a applied voltage of 1.5 V or 1 mA cm^−2^ at a applied voltage of 1 V, the total cost can even beat the current market price of Li ion extraction from minerals and brines by traditional process, even before considering the additional profits from the treatment of waste battery.

In this study, 0.1 M of Li ion concentration close to the Li content level of waste battery is primarily selected for experimental demonstration, while this is not the limitation of PEC Li metal extraction technology. The proposed PEC extraction system can also be coupled with other separation or extraction process (i.e., electrodialysis (ED)) [57] to concentrate the low-concentration Li sources (i.e., the electrolyte effluent from the Li metal extraction cell) into target concentrations. Following the TEA, the cost breakdown demonstrates that the profitability of the PEC process could show potentially viable by including an extra concentration step. For application to a source with an even lower Li concentration (such as seawater), the operational cost of concentrating step could become dominant and its cost could not be compensated from the proposed PEC extraction system. In this situation, a cost-efficient technique to concentrate Li sources will be explored and developed, and some routes (i.e., government intervention) would be necessary to help utilize these low-concentration Li sources to ensure efficient extraction of Li metals [58]. The overall analysis reinforces the potential of sustainable PEC extraction process as a feasible alternative to local treatment plants of waste batteries and the traditional processes (such as evaporation concentration, see Fig. 1).

## Conclusions

Waste batteries can serve as one of the next generation of Li resources for the future Li mining industry. In this work, we propose a solar-powered method for selectively and efficiently extracting the Li metals from the multi-cation electrolytes by the difference of redox potential. Through screening the reaction conditions (i.e., electrode materials and electrolyte concentrations) and assembling the two-electrode PEC devices, the designed PEC system not only can produce the high-purity Li metals from the multi-cation electrolytes but also shows potential for purifying the waste electrolytes and recycling the other metals (i.e., Fe, Co and Ni). Using a coplanar ATOS photocathode-TiO_2_ photoanode PEC device, our prototypic setup exhibits an acceptable extraction rate of ~ 1.38 g h^−1^ m^−2^, an excellent FE of 90.7% and a high production purity of 99.5% at overall applied potential of 2.0 V under 1 sun illumination. We also demonstrate the techno-economic feasibility of this PEC system, which could offer a more sustainable alternative for Li metal extraction and contribute to the treatment of waste batteries. In this work, we present one such pathway and anticipate future investigations to further develop the PEC system in a practical recycle and extraction of metal resources.

## Supplementary Information

Below is the link to the electronic supplementary material.

Supplementary file1 (MP4 1139 KB)

Supplementary file2 (DOCX 12131 KB)
