# Supplementary material for: Recycling of High-Purity Lithium Metal from Waste Battery by Photoelectrochemical Extraction at Ultralow Overall Potential
Source: Nanomicro Lett. 2026 Jan 14;18:117. doi: 10.1007/s40820-025-01958-z (PMC12799832; doi:10.1007/s40820-025-01958-z)
Supplement: Supplementary file 2 — Supplementary file2 (DOCX 12131 KB) [file 40820_2025_1958_MOESM2_ESM.docx]

Supporting Information for

**Recycling of High-Purity Lithium Metal from Waste Battery by Photoelectrochemical Extraction at Ultralow Overall Potential**

Longfei Yang^1,2†^, Chao Huang^1†^, Yanhong Lyu^1†^*, Dawei Chen^1,2^*, Aibin Huang^3,4^ and Jianyun Zheng^1^*

^1^ State Key Laboratory of Chemo and Biosensing, College of Chemistry and Chemical Engineering, International Joint Lab of Energy Electrochemistry of the Ministry of Education, Hunan University, Changsha 410082, P. R. China

^2^ College of Material Science and Engineering, Qingdao University of Science and Technology, Zhengzhou Road 53, Qingdao, 266042, P. R. China

^3^ State Key Laboratory of High Performance Ceramics and Superfine Microstructure, Shanghai Institute of Ceramics, Chinese Academy of Sciences, Shanghai 200050, P. R. China

^4^ Center of Materials Science and Optoelectronics Engineering, University of Chinese Academy of Sciences, Beijing, 100049, P. R. China

†Longfei Yang, Chao Huang, and Yanhong Lyu contributed equally to this work

*Corresponding authors. E-mail: [lvyanhong603@163.com](mailto:lvyanhong603@163.com) (Yanhong Lyu); [daweichen@qust.edu.cn](mailto:daweichen@qust.edu.cn) (Dawei Chen); [jyzheng@hnu.edu.cn](mailto:jyzheng@hnu.edu.cn) (Jianyun Zheng)

**Supplementary Figures and Tables**


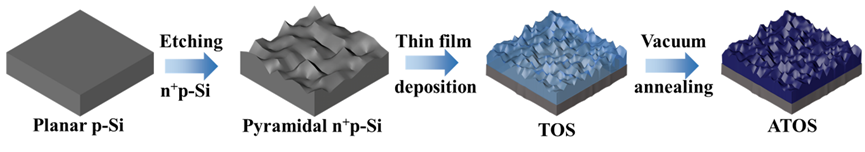


**Fig. S1** | A scheme for fabrication of the ATOS photocathode

Taking TiO_2_ cocatalyst layer as an example, the preparation of Si-based hierarchical photocathode for PEC Li metal extraction is displayed in **Fig.** S1. A planar p-type Si wafer underwent an alkaline etching and a phosphorization treatment to obtain the n^+^p-Si light absorber with pyramidal surface, as usually served in photovoltaic industry. A cocatalyst TiO_2_ layer was deposited on the n^+^p-Si surface using magnetron sputtering technology. Further annealing treatment was implemented at 100 °C to improve the structure and performance of cocatalyst layer for PEC Li metal extraction. The pristine TiO_2_/n^+^p-Si, WO_3_/n^+^p-Si and MoO_3_/n^+^p-Si photocathodes are labeled as TOS, WOS and MOS, while the corresponding annealing photocathodes are simply marked as ATOS, AWOS and AMOS, respectively. In addition, other deposition conditions and annealing temperatures were also investigated to tune the thickness and crystallinity of cocatalyst layer and optimize the PEC extraction performance of Si-based photocathodes.

**
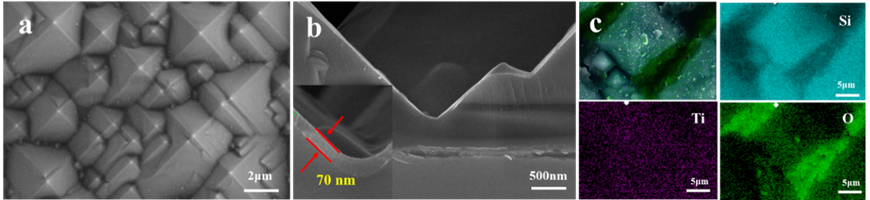
**

**Fig. S2** | FESEM images and EDS mapping of ATOS photocathode: (**a**) Top-view FESEM image of the ATOS photocathode, (**b**) Cross-sectional FESEM image of the ATOS photocathode, (**c**) EDS mapping of the ATOS photocathode showing Si, Ti, and O elements

**
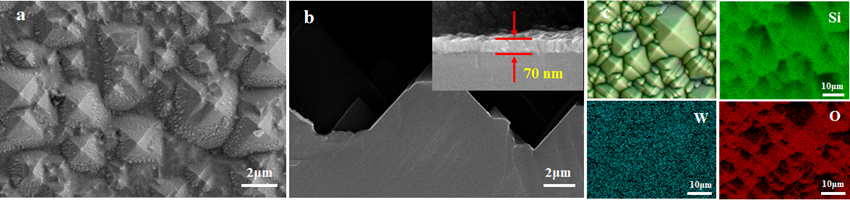
**

**Fig. S3** | FESEM images and EDS mapping of AWOS photocathode: (**a**) Top-view FESEM image of the AWOS photocathode, (**b**) Cross-sectional FESEM image of the AWOS photocathode, (**c**) EDS mapping of the AWOS photocathode showing Si, W, and O elements


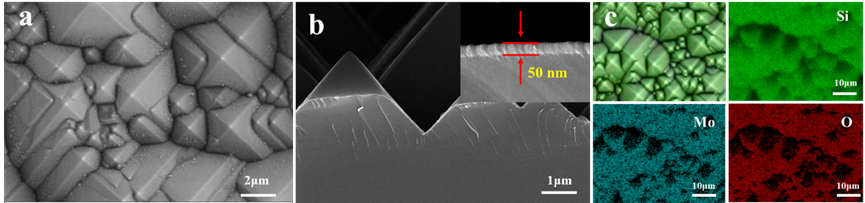


**Fig. S4** | FESEM images and EDS mapping of AMOS photocathode: (**a**) Top-view FESEM image of the AMOS photocathode, (**b**) Cross-sectional FESEM image of the AMOS photocathode, (**c**) EDS mapping of the AMOS photocathode showing Si, Mo, and O elements.

As shown in **Fig.** S2-S4, the cocatalyst layer with the thickness of 50-70 nm is conformal by pyramidal geometry coating on the surface of n^+^p-Si


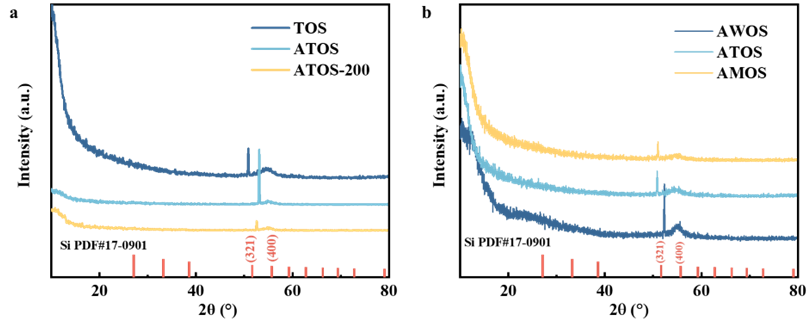


**Fig. S5** | (**a**) XRD patterns of TOS, ATOS, and ATOS-200 photocathodes. (**b**) XRD patterns of AWOS, ATOS, and AMOS photocathodes.

No diffraction peak was observed in ATOS, AWOS, AMOS and other photocathodes with different annealing temperatures, implying the presence of nano-grains in these cocatalyst layers. Conventionally, higher annealing temperature can result in bigger nano-grains in the cocatalyst layer


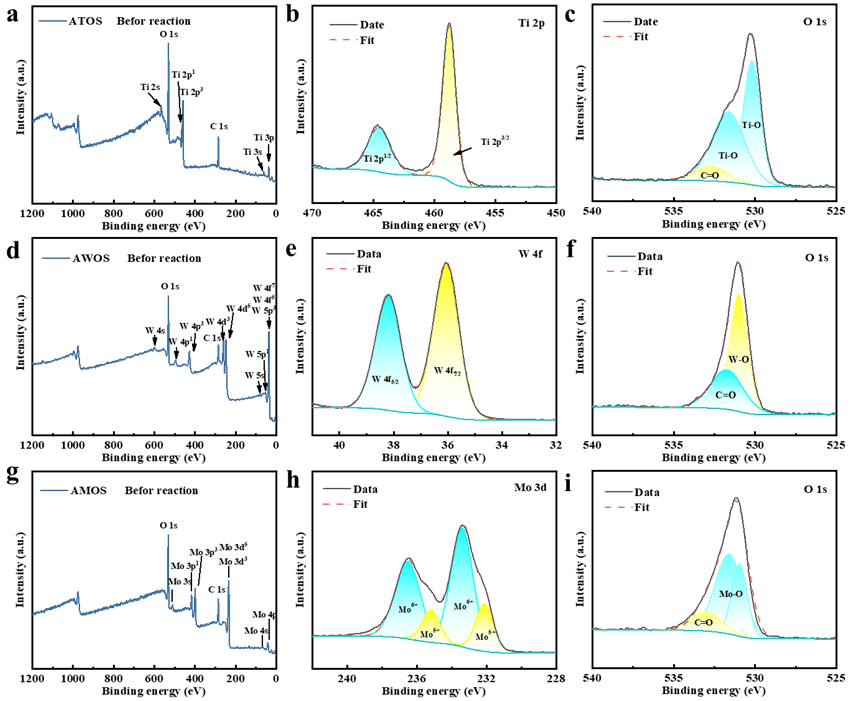


**Fig. S6** | XPS spectra of Si-based photocathodes before reaction: (**a-c**) full spectrum, Ti 2p, and O 1s analysis of ATOS photocathode, respectively; (**d-f**) full spectrum, W 4f, and O 1s analysis of AWOS photocathode, respectively; (**g-i**) full spectrum, Mo 3d, and O 1s analysis of AMOS photocathode

In addition to the C contaminants, **Fig.** S6a, S6d and S6g show the signal of Ti, W and Mo elements, respectively. As shown in **Fig.** S6b, S6e and S6h, Ti 2p_3/2_, W 4f_7/2_ and Mo 3d_5/2_ peaks at 458.8, 36.1 and 233.3 eV were assigned to Ti^4+^, W^6+^ and Mo^6+^, respectively. These results suggest the successful preparation of TiO_2_, WO_3_ and MoO_3_ film on the n^+^p-Si.


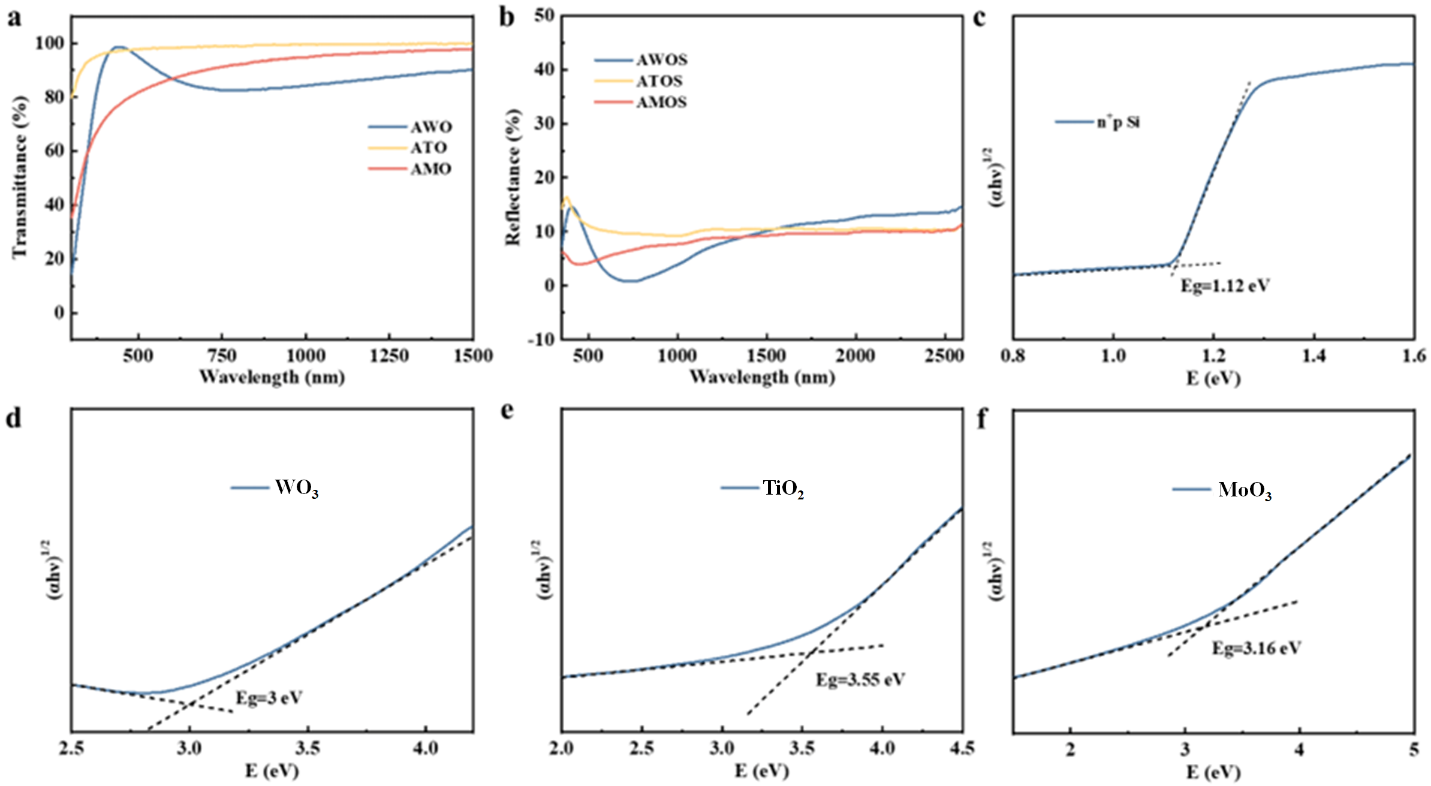


**Fig. S7** | Optical properties of Si-based photocathodes. (**a**) Transmittance measured for ATO, AWO and AMO layers deposited on glass substrates. (**b**) Total hemispherical optical reflectance of ATOS, AWOS and AMOS photocathodes measured in air. Absorption coefficient of the photocathodes as a function of incident photon energy: (**c**) n^+^p silicon, (**d**) WO_3_ cocatalyst layer, (**e**) TiO_2_ cocatalyst layer, and (**f**) MoO_3_ cocatalyst layer.

In the optical measurements (**Fig.** S7), ATOS, AWOS and AMOS showed similar optical diffuse reflectance spectra with ~10% of reflectance in the range from 320 to 2600 nm, and meanwhile the annealing cocatalyst layers deposited on BK7 glass wafer via the same preparation conditions of the photocathodes had highly transparent (over 80%). According to the indirect allowed transition, the band gap values of n^+^p-Si, TiO_2_, WO_3_ and MoO_3_ are about 1.12, 3.55, 3.00 and 3.16 eV, respectively, which are in line with the previous reports.


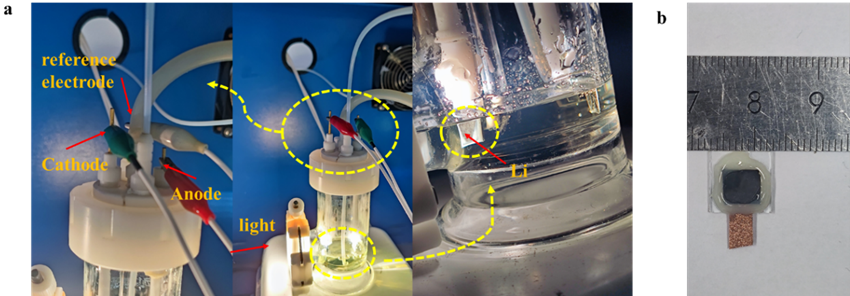


**Fig. S8** | (**a**) PEC reaction setup for Li metal extraction in three-electrode system, including light source and reaction cell. The right **Fig.** shows Li metals extracted from the surface of Si-based photocathode during the reaction process. (**b**) Photograph of Si-based photocathode

In this three-electrode PEC system, Si-based photocathode, Pt and Ag/AgCl were used as the cathode, anode and reference electrode, respectively. Solar illumination was provided by a solar simulator (fiber optic source, FX300) under AM 1.5G conditions at 100 mW·cm^-2^. To avoid the disturbance of dissolved oxygen O_2_ in the electrolytes and facilitate the mass transfer on the photocathode surface, the Ar stream as protective gas was continuously bubbled in the electrolytes of reaction cell. In addition, the entire back side and partial front side of Si-based photocathode was encapsulated by the epoxy to prevent the enhancement of light scattering (see **Fig.** S8b).


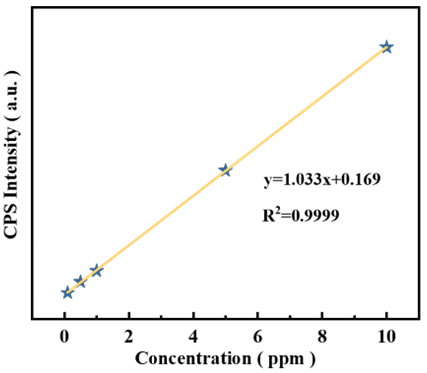


**Fig. S9** | ICP calibration curve for known Li⁺ concentrations

The standard LiClO_4_ solutions with known concentrations were prepared, and the calibration curve was obtained by ICP-MS (**Fig.** S9). In the calibration curve, y = 1.033x + 0.169 and R^2^ = 0.9999 (y, x and R^2^ denote as CPS intensity, Li^+^ concentration and degree of fitting, respectively).


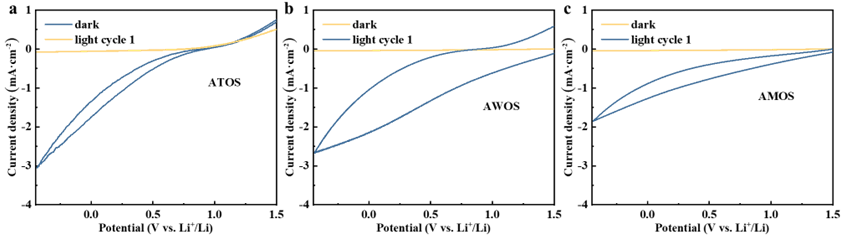


**Fig. S10** | CV curves of various photocathodes measured in 0.1 M LiClO_4_-PC solutions at a scan rate of 0.05 V·s^-1^ under AM 1.5 G simulated sunlight (100 mW‧cm^-2^): (**a**) ATOS photocathode, (**b**) AWOS photocathode, and (**c**) AMOS photocathode.

The CV analysis of the Si-based photocathodes were applied to demonstrate the rate of Li^+^ reduction (**Fig.** S10). The dark reduction current was negligible, implying that the driving force of the Li-extraction reaction is from the light. Under illumination, the obvious photocurrents were observed in the ATOS, AWOS and AMOS photocathodes. In comparison to AWOS annd AMOS photocathodes, ATOS photocathode showed a fast reduction of Li^+^ with the reduction rate of ~3 mA cm^-2^ at -0.4 V vs. Li^+^/Li swept forward to more negative; similarly fast is the reoxidation of the reduced Li species on the return cycle.


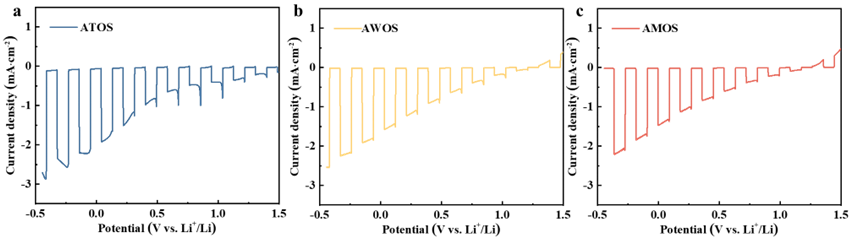


**Fig. S11** | Chopped LSV curves of various photocathodes measured in 0.1 M LiClO_4_-PC solutions at a scan rate of 0.03V s^-1^ under AM 1.5 G simulated sunlight (100 mW‧cm^-2^): (**a**) ATOS photocathode, (**b**) AWOS photocathode, and (**c**) AMOS photocathode

Compared to AWOS and AMOS photocathodes, ATOS photocathode showed a higher photocurrent density, similar to the results of their CV curves.


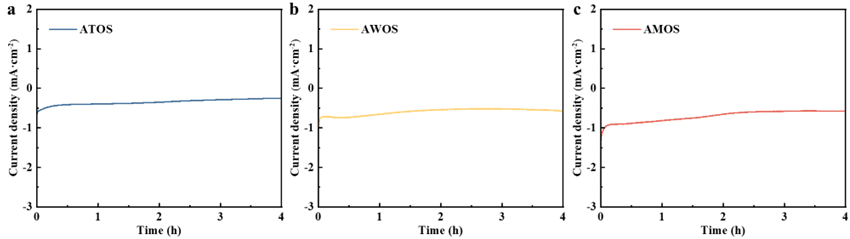


**Fig. S12** | The photocurrent density (*J*)-time (*t*) curves of ATOS (**a**), AWOS (**b**) and AMOS (**c**) photocathodes obtained in 0.1 M LiClO_4_-PC solutions under 1 sun irradiation at 0.056 V vs. Li^+^/Li for 4 hours

**Figure** S12 shows the *J*-*t* curves of the Si-based photocathodes at applied potential of 0.056 V vs. Li^+^/Li for 4 hours. During the 4-hours extraction process, the *J*-*t* curves of all the photocathodes were relatively constant, indicating a stable operation for PEC Li extraction.


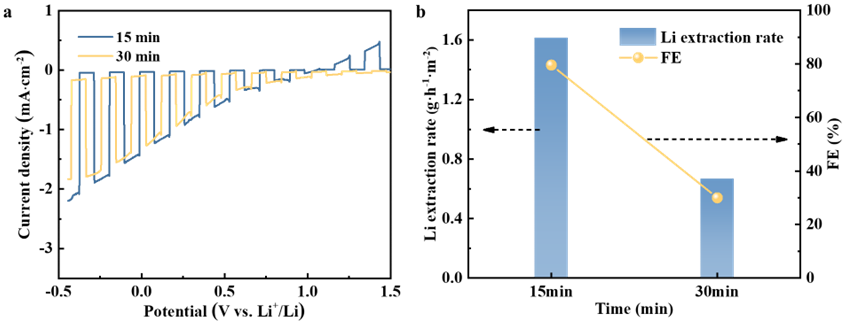


**Fig. S13** | PEC performance of ATOS at 15- and 30-min TiO_2_ layer: (**a**) chopped LSV curves; (**b**) corresponding extraction rate and FE of Li metals

As shown in **Fig.** S13b, ATOS with 15-min TiO_2_ layer had the better performance of Li metal extraction with higher extraction rate and FE than that with 30-min TiO_2_ layer.


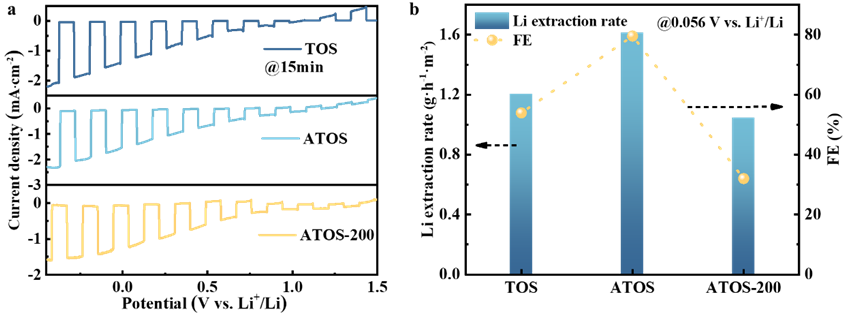


**Fig. S14** | PEC performance of TOS, ATOS and ATOS-200 at 0.056 V vs. Li^+^/Li in 0.1 M LiClO_4_-PC solutions: (**a**) chopped LSV curves; (**b**) corresponding extraction rate and FE of Li metals

As shown in **Fig.** S14b, ATOS had the optimal performance of Li metal extraction with the highest extraction rate and FE.


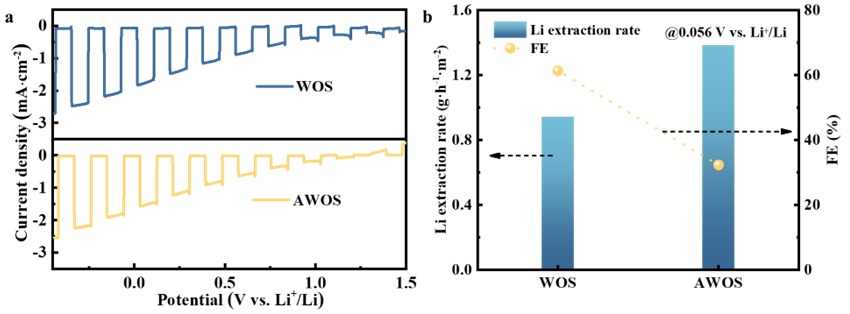


**Fig. S15** | PEC performance of WOS and AWOS at 0.056 V vs. Li^+^/Li in 0.1 M LiClO_4_-PC solutions: (**a**) chopped LSV curves; (**b**) corresponding extraction rate and FE of Li metals

As shown in **Fig.** S15b, AWOS had the better performance of Li metal extraction with the higher extraction rate and FE than WOS.


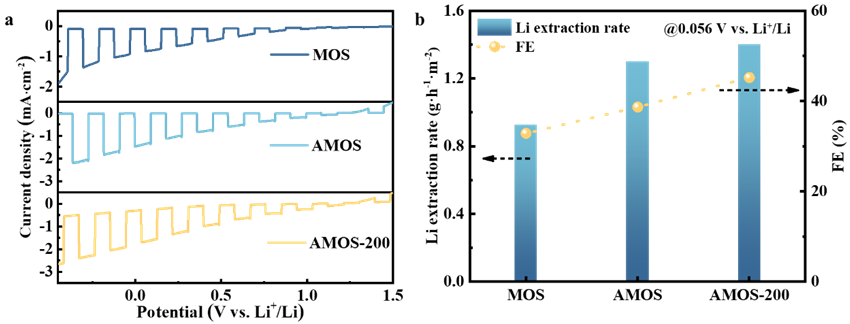


**Fig. S16** | PEC performance of MOS, AMOS and AMOS-200 at 0.056 V vs. Li^+^/Li in 0.1 M LiClO_4_-PC solutions: (**a**) chopped LSV curves; (**b**) corresponding extraction rate and FE of Li metals


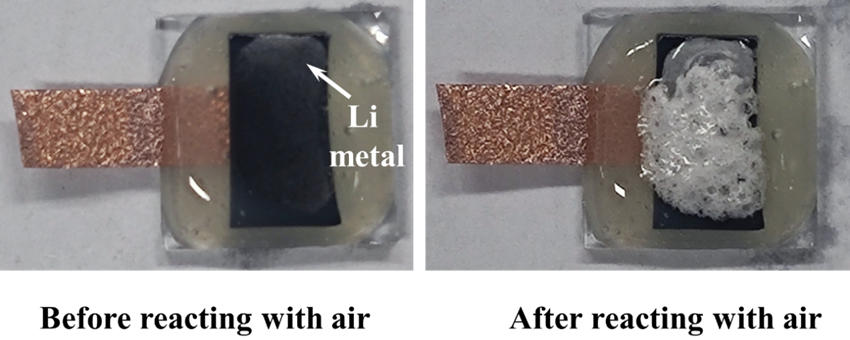


**Fig. S17** | Photographs of the post-electrolysis ATOS photocathode before and after exposing in the air

It can be clearly observed that some white particles were deposited on the surface of ATOS photocathode, which happened a sharp reaction with the air to produce a mass of bubbles.


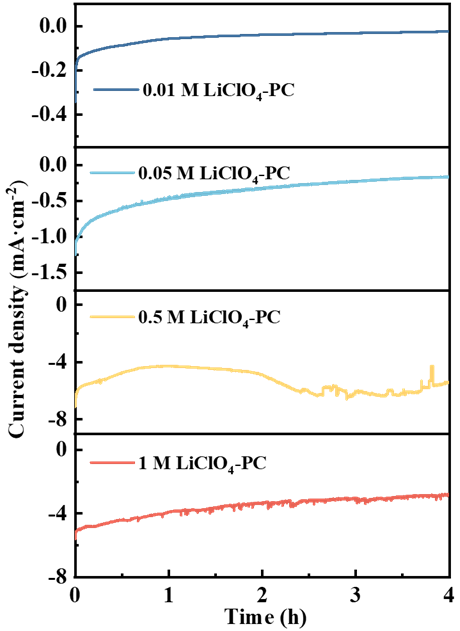


**Fig. S18** | *J*-*t* curves of ATOS photocathodes in the electrolytes with different LiClO_4_ concentrations during a 4-h reaction at 0.056 V vs. Li^+^/Li


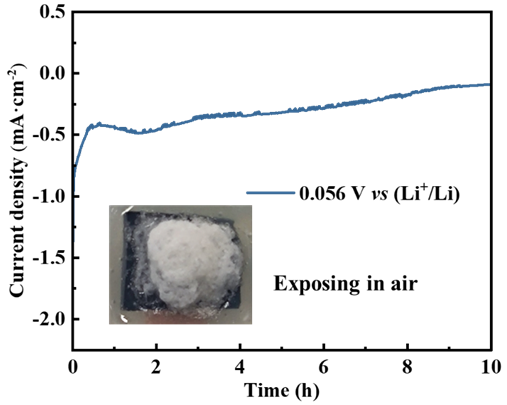


**Fig. S19** | The *J*-*t* curve of ATOS photocathode in the 0.1 M LiClO_4_-PC electrolytes at 0.056 V vs. Li^+^/Li under 1 sun illumination during the 10-h reaction. The inset shows the surface changes of the reacted ATOS photocathode after exposing in the air


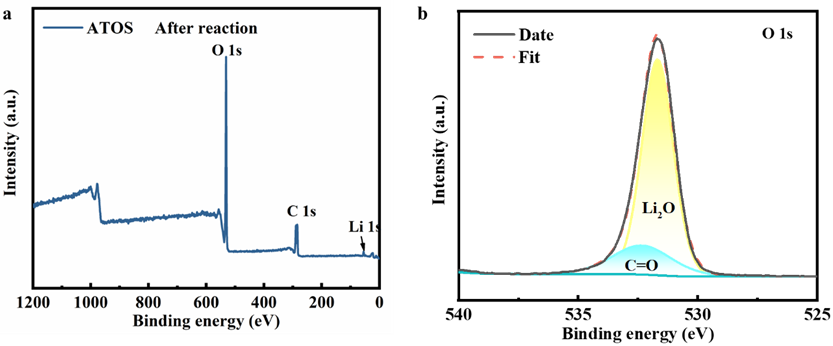


**Fig. S20** | XPS spectra of the ATOS photocathode after PEC Li metal extraction: (**a**) full spectrum, and (**b**) O 1s spectrum

As shown in **Fig.** S20a, there was no Ti and Si signal in the ATOS photocathode, implying that the Li metal layer was enough thick.


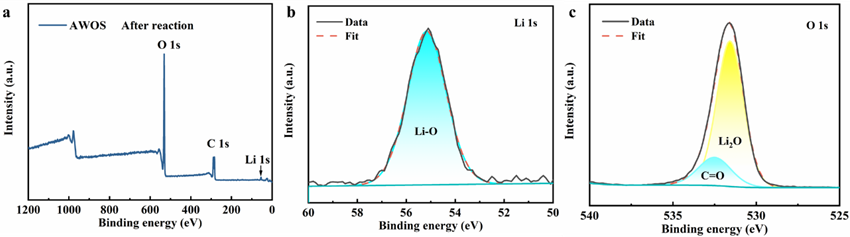


**Fig. S21** | XPS spectra of the AWOS photocathode after PEC Li metal extraction: (**a**) full spectrum, (**b**) Li 1s spectrum, and (**c**) O 1s spectrum


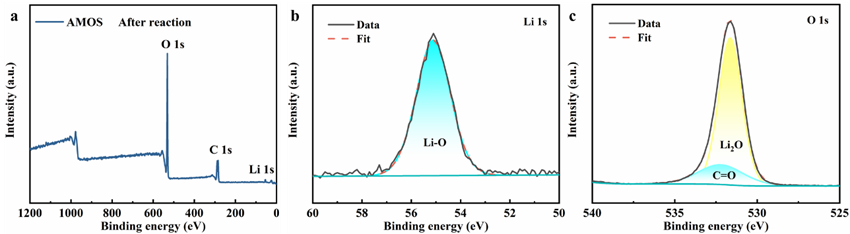


**Fig. S22** | XPS spectra of the AMOS photocathode after PEC Li metal extraction: (**a**) full spectrum, (**b**) Li 1s spectrum, and (**c**) O 1s spectrum


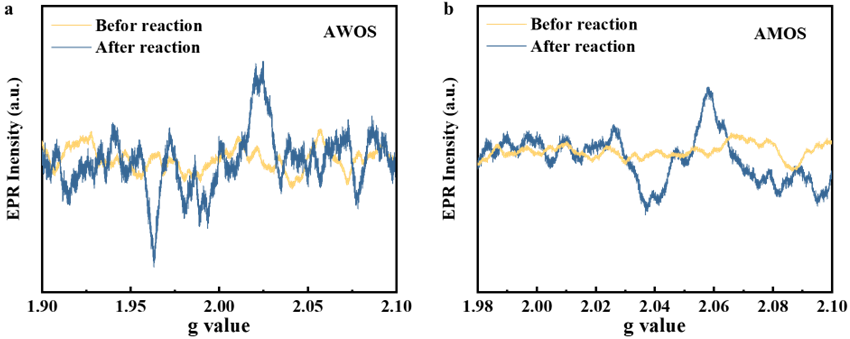


**Fig. S23** | Comparison of EPR spectra before and after PEC Li metal extraction in 0.1 M LiClO_4_-PC electrolytes at 0.056 V vs. Li^+^/Li potential for 4 h: (**a**) AWOS, and (**b**) AMOS


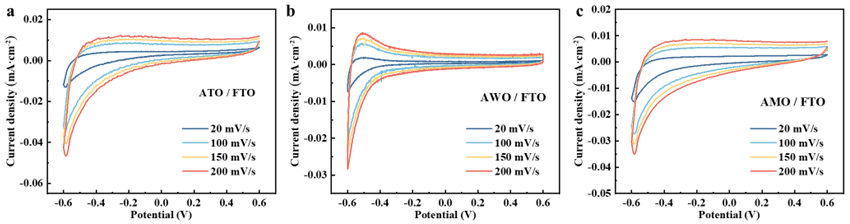


**Fig. S24** | CV curves of (**a**) ATO, (**b**) AWO, and (**c**) AMO layers on FTO wafers recorded in 1.0 M LiClO_4_-PC solutions. The scan rates are 20, 100, 150, and 200 mV·s⁻¹. The sedimentary conditions are the same as those of the n^+^p-Si

Based on the relationship between the CV scan rate and peak current (**Fig.** 2e), the Li⁺ diffusion coefficients for the TiO_2_, WO_3_, and MoO_x_ layers can be calculated using the Randles-Sevcik equation **[**1]:

$J_{pc}=2.69\times10^{5}n^{\frac{3}{2}}D^{\frac{1}{2}}\nu^{\frac{1}{2}}C_{0}$ (S1) where *J*_pc_, *n*, *D*, *ν* and *C*_0_ are the peak current density, number of transferred charges, diffusion coefficient of Li ions, potential scan rate and concentration of Li ions, respectively. A linear relationship between the peak current density and the square root of the scan rate indicates that the Li⁺ insertion process is diffusion-controlled. The Li⁺ diffusion coefficients (*D*_Li_) for the TiO_2_, WO_3_, and MoO_x_ layers were calculated according to Equation S2, as listed in Table S1. Compared to the WO₃ and MoOₓ layers, the TiO₂ layer exhibited a higher *D*_Li_ value.


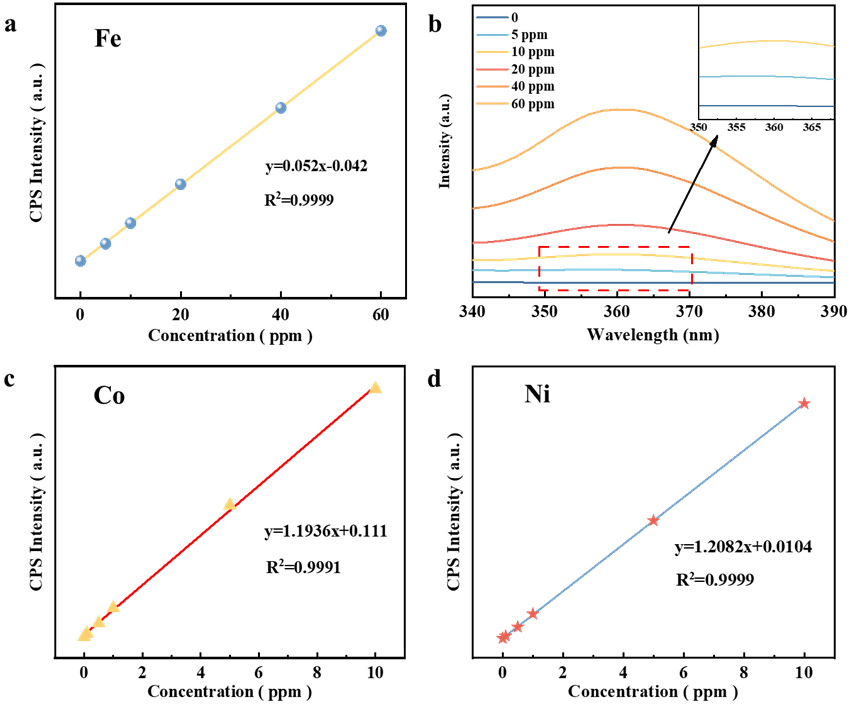


**Fig. S25** | Calibration curves for known concentrations of Fe, Co, and Ni: (**a**) calibration curve for known concentrations of Fe ions, (**b**) UV-vis absorbance curve for Fe ions at 340-390 nm, (**c**) ICP calibration curve for known concentrations of Co ions, (d) ICP calibration curve for known concentrations of Ni ions.


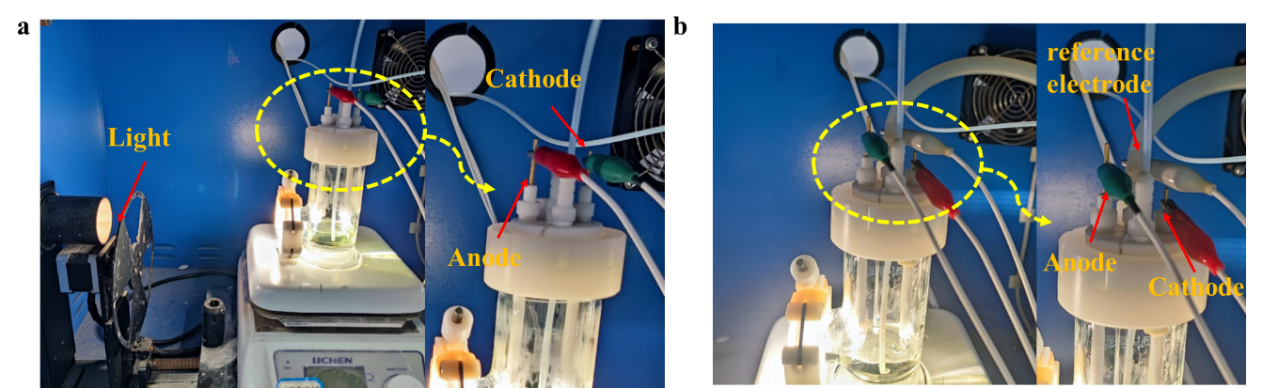


**Fig. S26** | Photographs of PEC reaction device for Fe, Co and Ni metal extraction, including a light source and a reaction cell: (**a**) two-electrode system; (**b**) three-electrode system

In the two-electrode system, the anode was TiO_2_ photoanode deposited on FTO, and the cathode was Ni foam. In the three-electrode system, the anode was TiO_2_ photoanode deposited on FTO, the cathode was Ni foam, and the reference electrode was Ag/AgCl.


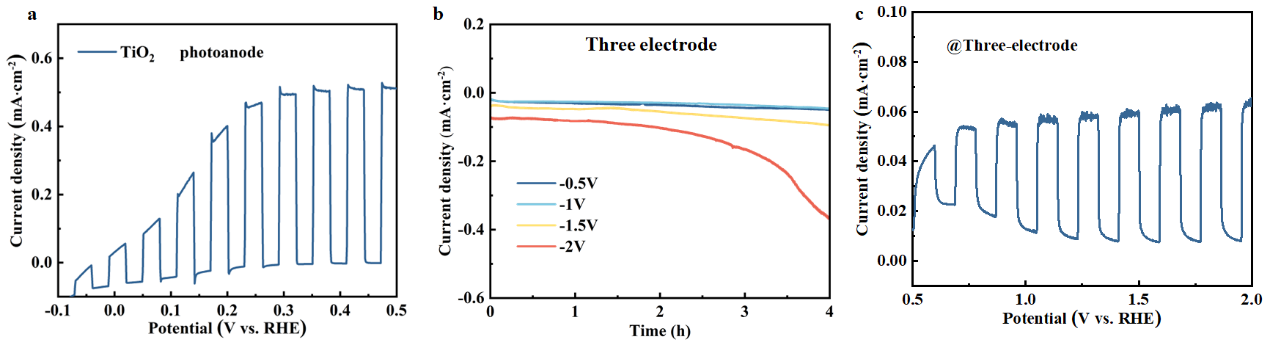


**Fig. S27** | PEC performance of TiO_2_ photoanode for Fe metal extraction in a three-electrode system: (**a**) chopped LSV curve in 1 M KOH solution for oxygen evolution reaction; (**b**) *J*-*t* curves at different potentials under 1 sun illumination in 1 mM FeCl_2_-PC solutions with TiO_2_ photoanode as counter electrode and Ni foam as working electrode; (**c**) chopped LSV curve in 1 mM FeCl_2_-PC solutions with TiO_2_ photoanode as working electrode


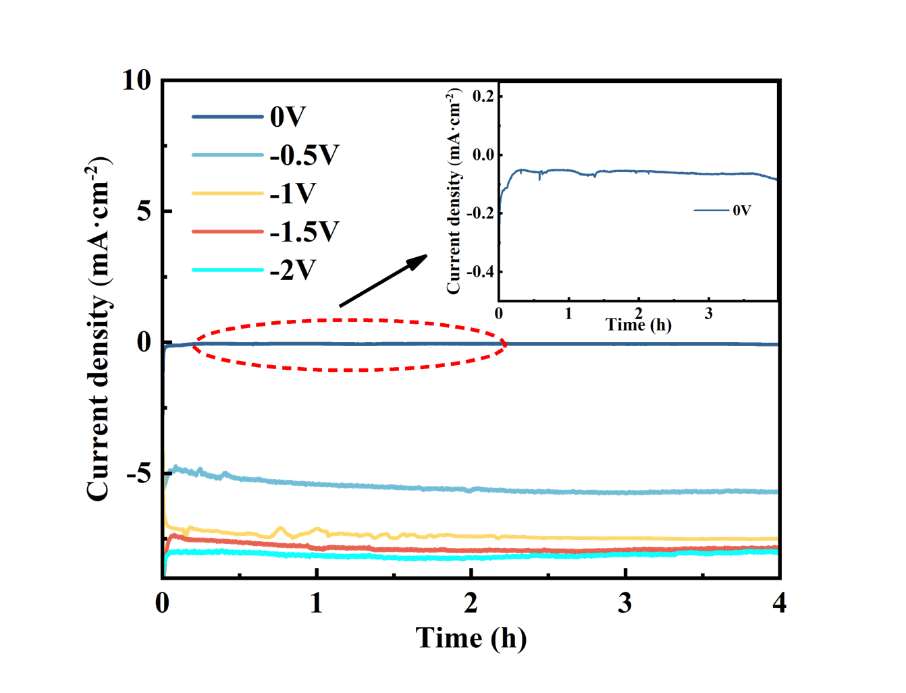


**Fig. S28** | *J*-*t* curves at different potentials in 1 mM FeCl_2_-PC solutions in a two-electrode system with TiO_2_ photoanode as counter electrode and Ni foam as working electrode. The inset shows the *J*-*t* curve at bias-free potential


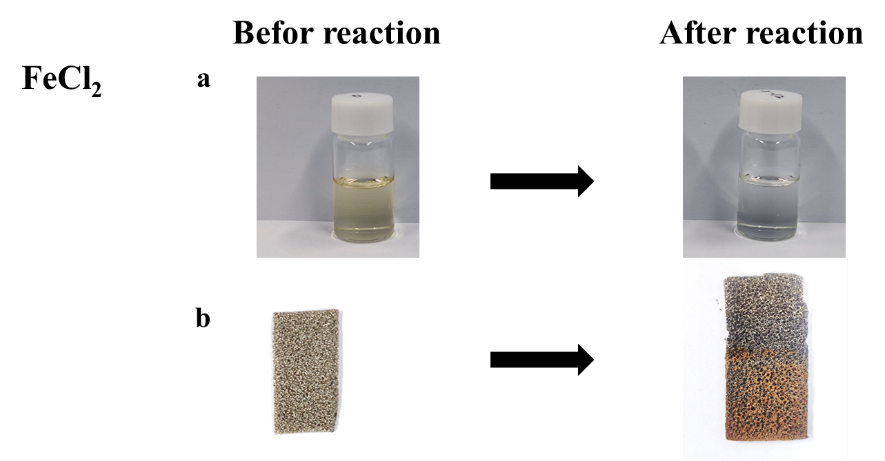


**Fig. S29** | Color changes of the electrolytes and Ni foam before and after 4-h PEC Fe metal extraction: (**a**) color change of the electrolytes from light yellow to colorless; (**b**) color change of the Ni foam from grey to brownish red


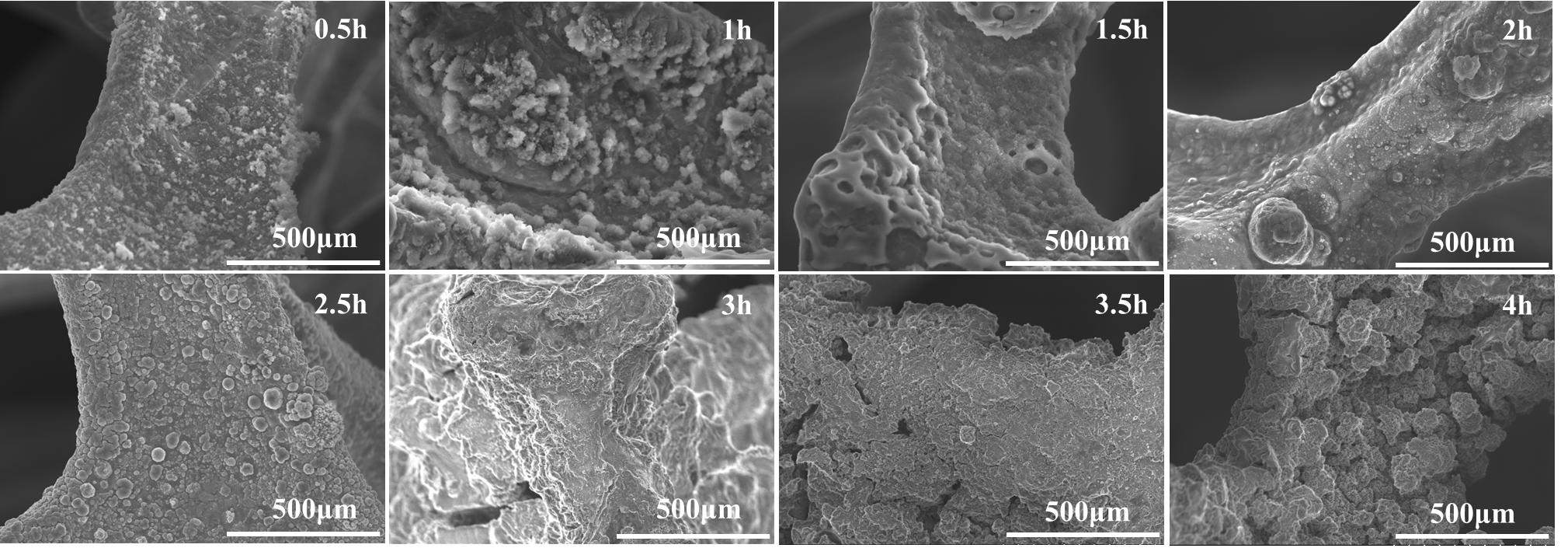


**Fig. S30** | Top-view FESEM images of the Ni foam surface at different extraction times in 1 mM FeCl₂-PC electrolytes in the two-electrode system

With the increase of extraction time, the structure of extracted Fe metals was initially dispersed as small particles to aggregated large particles, then to discontinuous islands, and finally accumulates into layers.


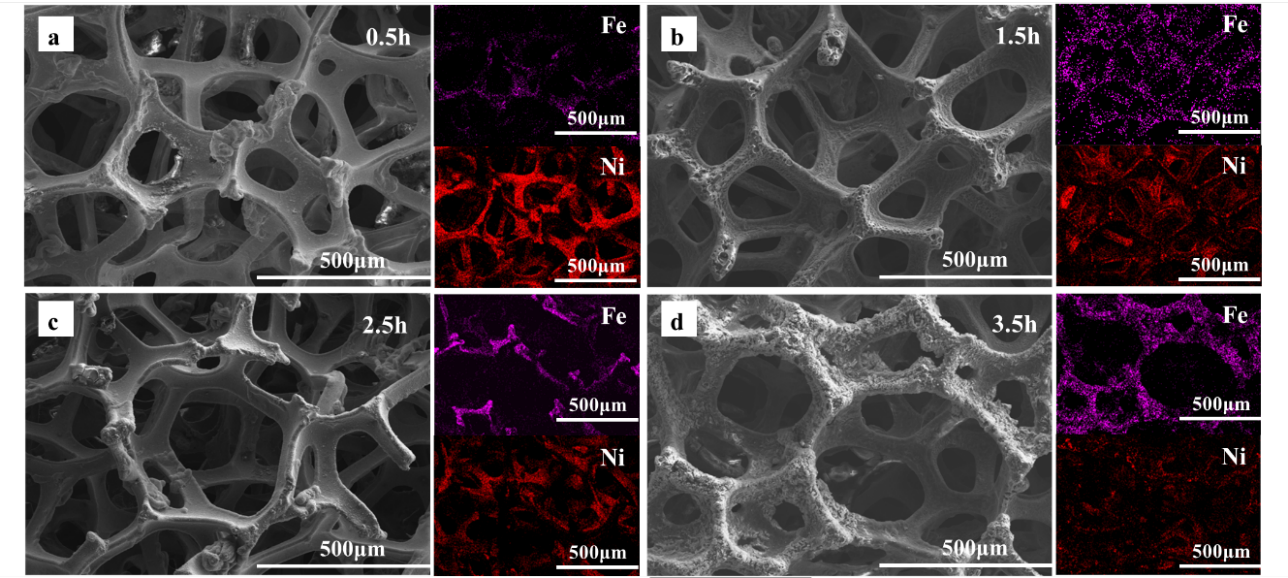


**Fig. S31** | FESEM and EDS mapping images of Ni foam surface after Fe metal extraction with different times: (**a**) 0.5h, (**b**) 1.5h, (**c**) 2.5h, and (**d**) 3.5h. The purple and red in these images represent Fe and Ni elements, respectively. With the extension of extraction time, the proportion of Fe on the surface of foam nickel gradually increased


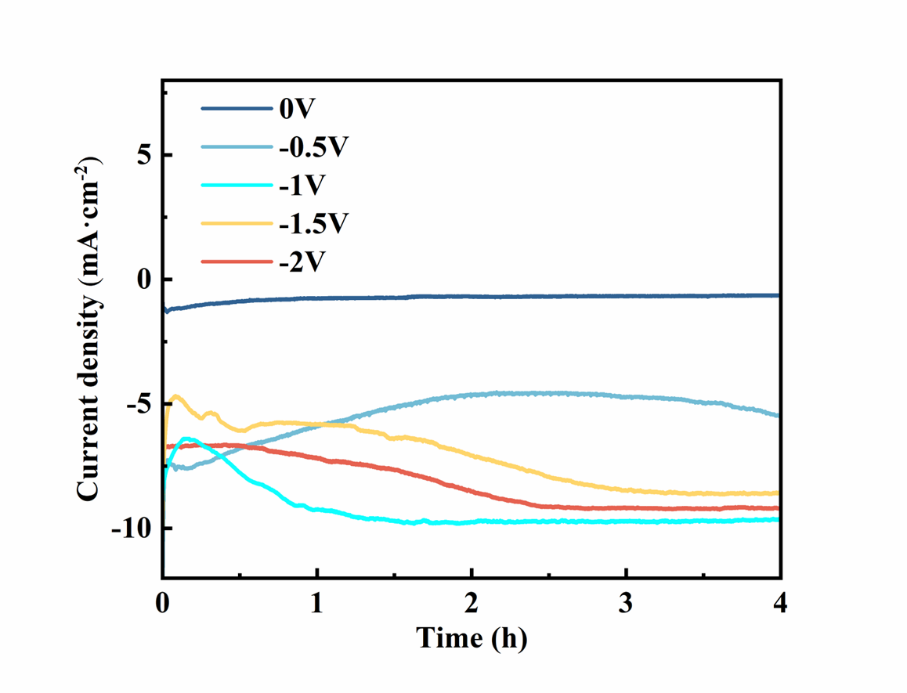


**Fig. S32** | *J*-*t* curves at different potentials in 1 mM CoCl_2_-PC solutions in a two-electrode system with TiO_2_ photoanode as counter electrode and Ni foam as working electrode


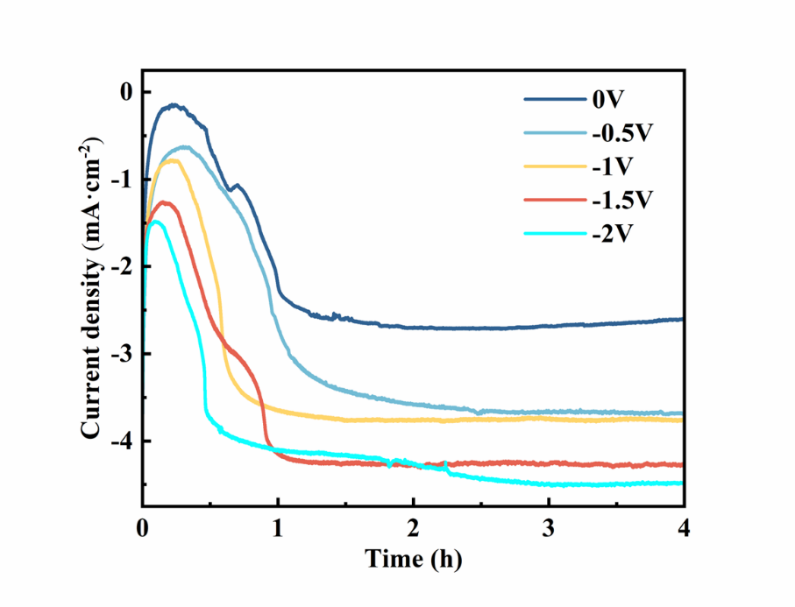


**Fig. S33** | *J*-*t* curves at different potentials in 10 mM Ni(ClO_4_)_2_ solutions in a two-electrode system with TiO_2_ photoanode as counter electrode and Ni foam as working electrode


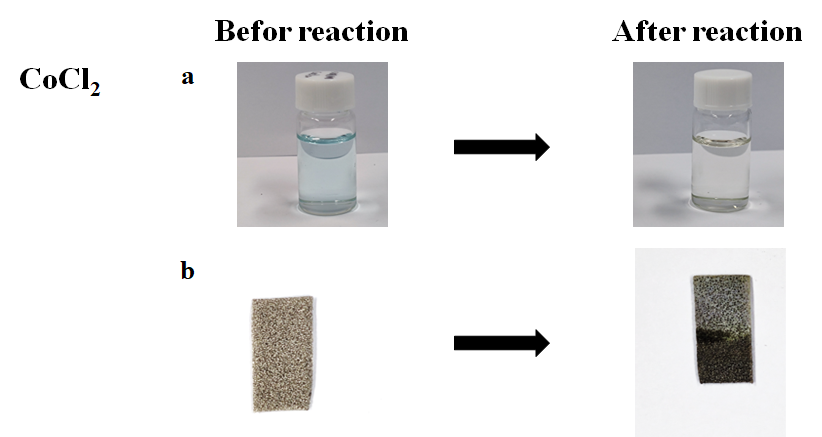


**Fig. S34** | Color changes of the electrolytes and Ni foam before and after 4-h PEC Co metal extraction: (**a**) color change of the electrolytes from blue to colorless; (**b**) color change of the Ni foam from grey to black


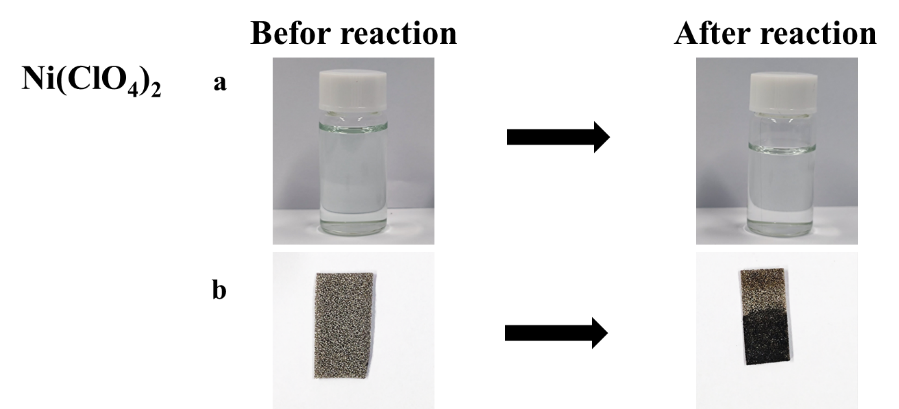


**Fig. S35** | Color changes of the electrolytes and Ni foam before and after 4-h PEC Ni metal extraction: (**a**) color change of the electrolytes from light green to colorless; (**b**) color change of the Ni foam from grey to black


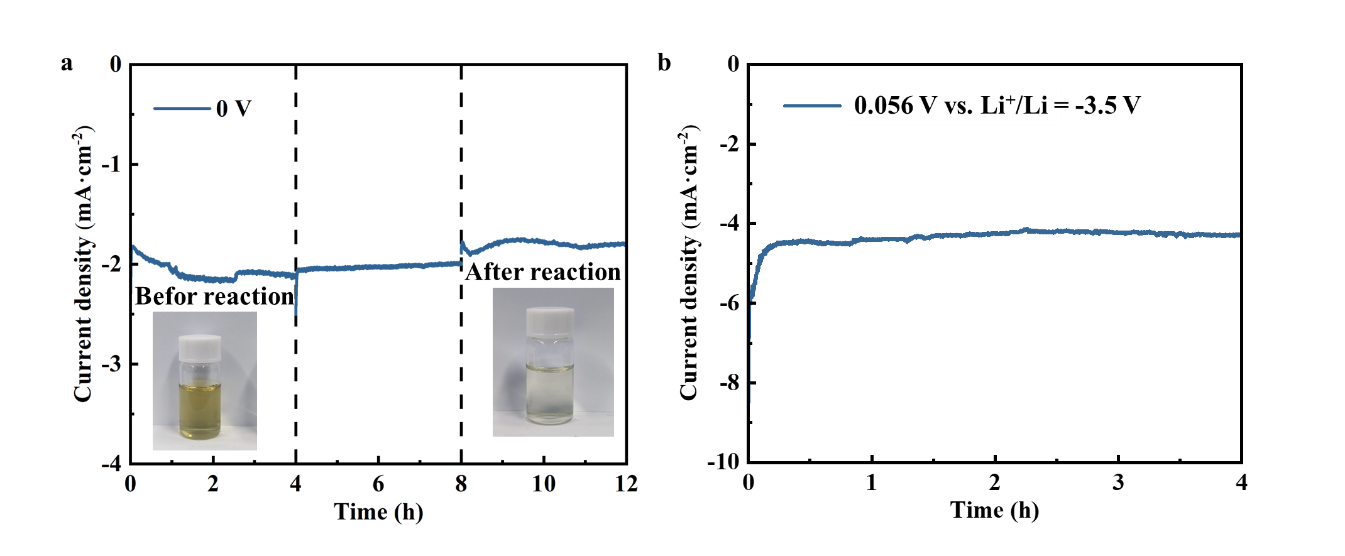


**Fig. S36** | *J*-*t* curves of the 12-h purifying process and 4-h Li metal extraction process in the two-electrode system in the mixed PC solutions of 0.1 M LiClO_4_, 1 mM FeCl_2_, 1 mM CoCl_2_, and 10 mM Ni(ClO_4_)_2_. (**a**) *J*-*t* curve for impure cation extraction including Fe, Co and Ni metals at 0 V with TiO_2_ photoanode and Ni foam. The insets are the color of the electrolytes before the reaction. (**b**) *J*-*t* curve for Li metal extraction with ATOS photocathode at 0.056 V vs. Li^+^/Li in the purified electrolytes under 1 sun illumination


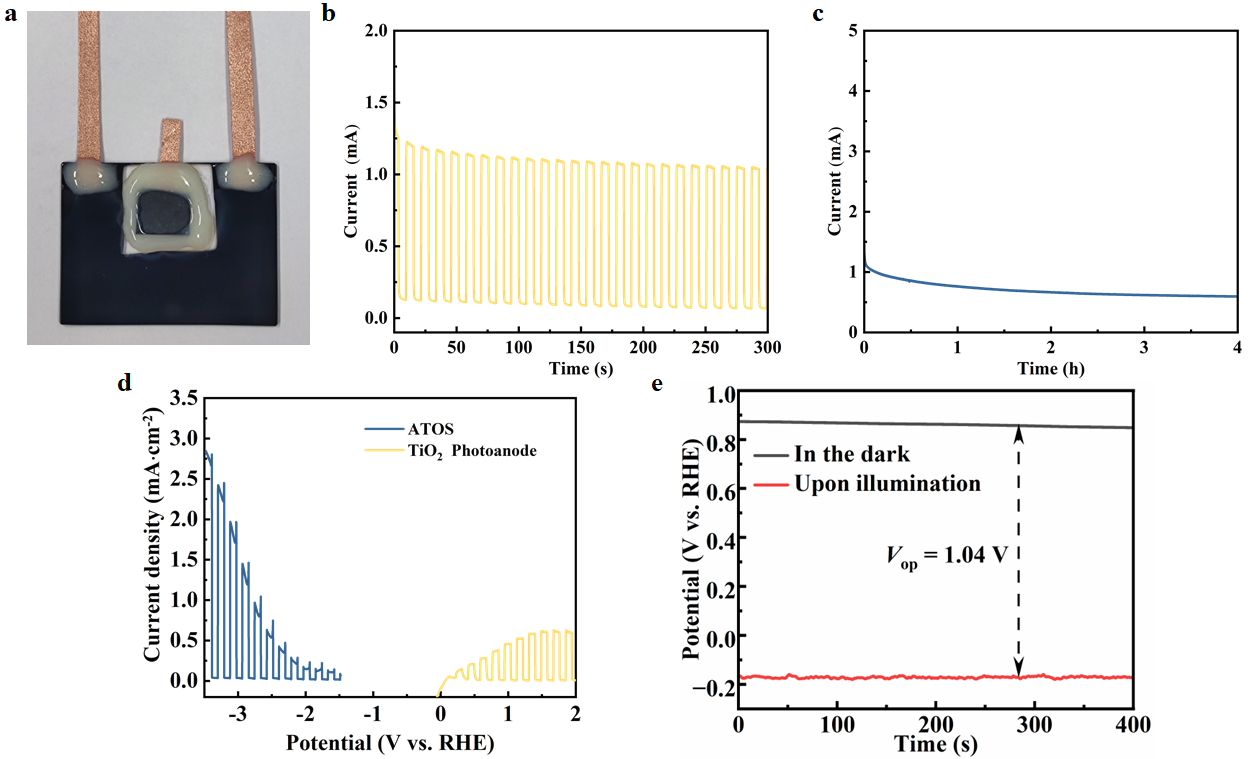


**Fig. S37** | ATOS-TiO_2_ device. (**a**) Photograph of the PEC device consisting of ATOS photocathode and TiO_2_ photoanode. (**b**) Chopped *J*-*t* curve of the device at an overall potential of 2 V in 0.1 M LiClO_4_-PC electrolytes under 1 sun illumination. (**c**) *J*-*t* curve of the device for Li metal extraction at an overall potential of 2 V in 0.1 M LiClO_4_-PC electrolytes under 1 sun illumination for 4 hours. (**d**) LSV curves of individual ATOS photocathode and TiO_2_ photoanode in a three-electrode system. The photocurrent sign of the photocathode is inverted to emphasize the photocurrent overlap and the value of applied bias. (**e**) Open-circuit potential (OCP) of TiO_2_ photoanode in 1 M KOH in the dark and upon illumination.

As shown in **Fig.** S37d, a visible photocurrent can be occurred between ATOS photocathode and TiO_2_ photoanode for PEC Li extraction and oxidation reactions at an overall potential of 2 V under 1 sun illumination. In addition, it can be found in **Fig.** S37e, the negative shift in OCP upon illumination indicates that TiO_2_ photoanode possesses n-type conductivity. The difference of OCP for TiO_2_ photoanode in the dark and upon illumination shows a high photovoltage (*V*_op_) of 1.04 V.


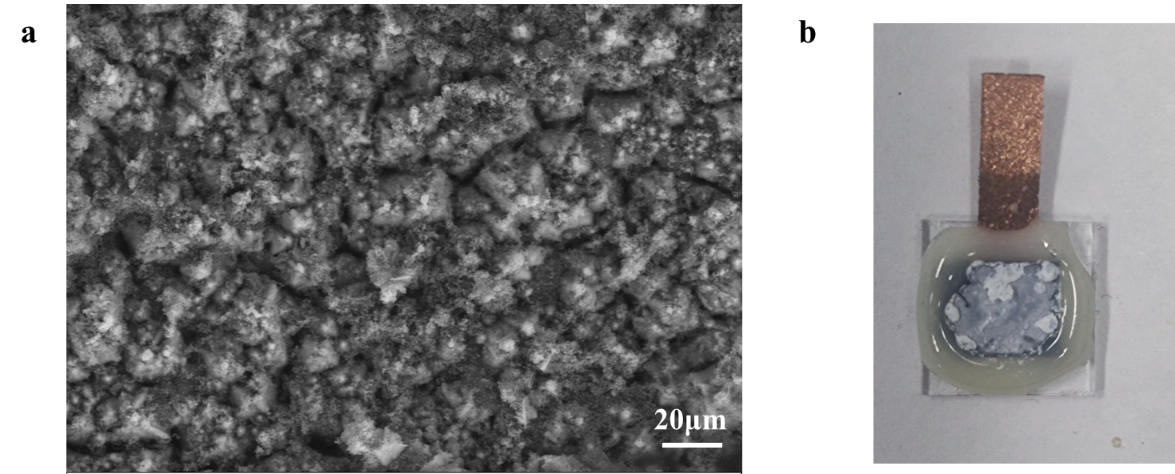


**Fig. S38** | (**a**) Top-view FESEM image of the ATOS photocathode surface after the 12-h purifying process and 4-h Li metal extraction process in the mixed PC solutions of 0.1 M LiClO_4_, 1 mM FeCl_2_, 1 mM CoCl_2_, and 10 mM Ni(ClO_4_)_2_. (**b**) Photograph of the ATOS photocathode after these processes


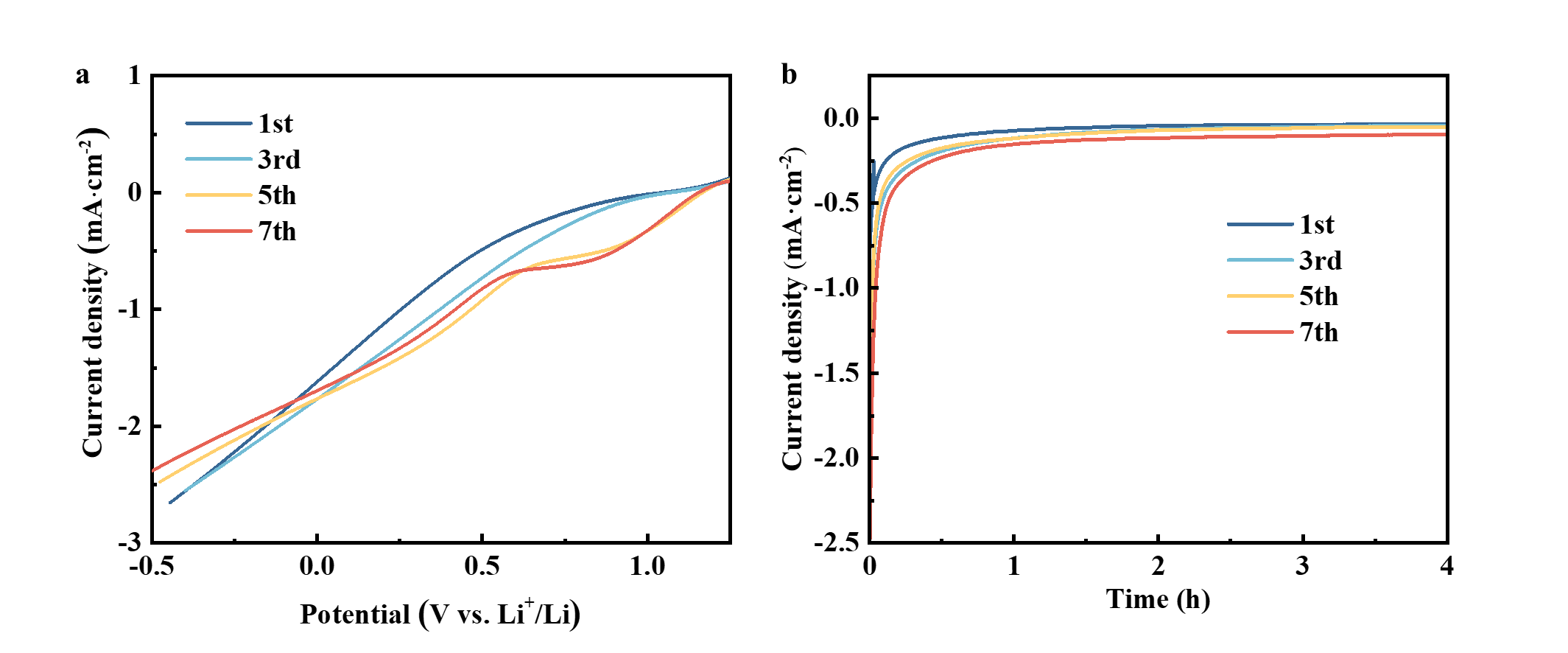


**Fig. S39** | (**a**) LSV curves and (**b**) *J*-*t* curves of different ATOS photocathodes for PEC Li metal extraction in the purified electrolytes at 0.056 V vs. Li^+^/Li after different cycles


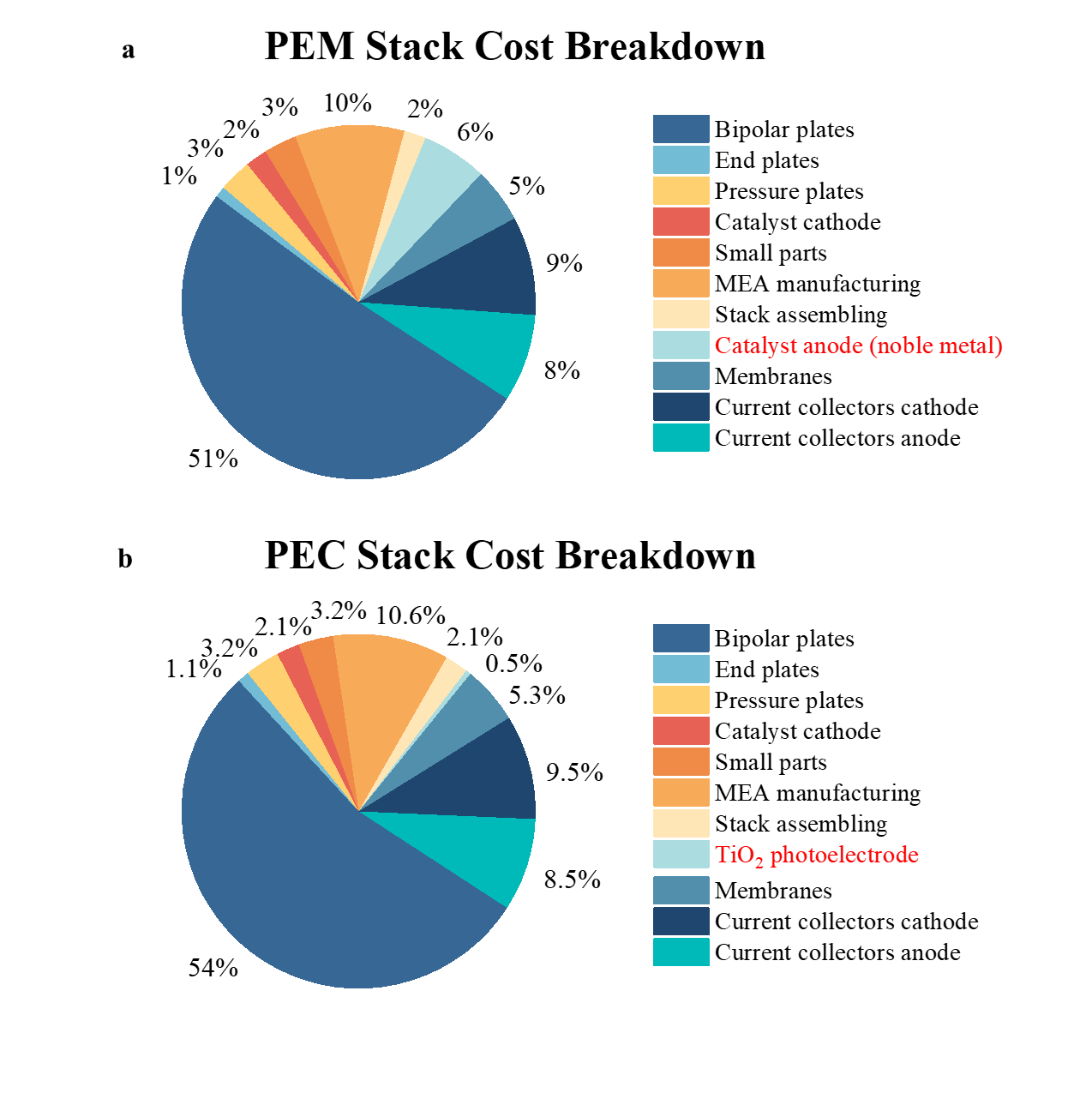


**Fig. S40** | (**a**) Stack cost breakdown for PEM electrolytic cell [2]. (**b**) Detailed cost breakdown for PEC electrolytic cell. The calculation for the PEC electrolytic cell was based on the E4tech/Element Energy report^2^, DOE H2A central grid electrolysis analysis,^3^ and previous models [4]. The cost of the anode was derived from the proportionate cost of the Ti target material consumed in the preparation of TiO_2_ film

**Table S1** Li-ion diffusion coefficients for TiO_2_, WO_3_, and MoO_x_ layers during Li insertion. The Li-ion diffusion coefficient is denoted as *D*_Li_, and the slope between *J*_pc_ and the scan rate is denoted as **Fig.** 2e.

| **Layer** | ***S*_l_ (mA·s^1 /2^·cm^-2^·V^-1/2^)** | ***D*_Li_ (cm^2^·s^-1^)** |
| --- | --- | --- |
| TiO_2_ | 0.0389 | 1.92×10^-14^ |
| WO_3_ | 0.0219 | 5.21×10^-15^ |
| MoO_x_ | 0.0281 | 8.58×10^-15^ |

**Table S2** Techno-economic analysis of PEC and Electrochemical Li metal Extraction

| **Sensitivity Parameters** | **This work** | **Electrochemistry^5^** |
| --- | --- | --- |
| **ECC^*1^** | 25.665 | 72 |
| **BOP^*2^** | 13.82 | 38.78 |
| **EC^*3^** | 0.2574 | 0.71 |
| **Main^*4^** | 8 | 22.44 |

*1. ECC represents Electrolytic cell cost

*2. BOP represents Balance of plant cost

*3. EC represents Electricity cost

*4. Main represents Maintenance cost

**Table S3** Input parameters for TEA model

| Li metal extraction yield | 1 kg day^–1^ |
| --- | --- |
| Operating time | 350 days year^–1^ |
| Electrolytic cell base cost^*1^ | 519.5 USD kW^–1^ |
| Balance of plant^*2^ | 35% of the electrolytic cell cost |
| Maintenance cost | 2.5% of the electrolytic cell cost |
| Plant lifetime | 20 years |
| Electric price^*3^ | 0.03 US$ kWh^–1^ |

*1. References to the E4tech/Element Energy report [2]

*2. References to the United States Department of Energy (DOE) H2A base model [3]

*3. References to the global weighted average levelized cost of energy for onshore wind electricity production [6]

**Note S1** **Techno-economic analysis of Li metal extraction**

The techno-economic analysis (TEA) in this study was developed and conducted based on previously reported models [4, 7, 8]. The anode is a TiO_2_ photoanode, with costs based on the proportion of Ti target material consumed in the preparation of TiO_2_ film. The comparative electrocatalytic model is the one from the E4tech/Element Energy report. Based on the performance in this study, the baseline parameters for the PEC extraction process were selected as follows: a current density of 0.65 mA‧cm^-2^, a total cell voltage of 2 V, and a FE of 90%. The detailed input parameters of the TEA model are listed in Table S2. In this model, transportation costs are ignored.

**Required power:**

Assuming the extraction rate of Li is 1 kg day^-1^, the total required current is:

$Current=1kg\cdot day^{-1}\times\frac{day}{86400s}\times\frac{kmol}{6.941}\times1e^{-}\times\frac{96485C}{mol}\times\frac{1}{90\%(FE)}=178.76A$ (S2)

The total area required for the electrolytic cell is:

$Area=\frac{178.76A}{0.00065A\cdot cm^{-2}}\times\frac{m^{2}}{10^{4}cm^{2}}=27.5m^{2}$ (S3)

The required power is calculated by P=UI

$Power=2V\times178.76A\times\frac{kW}{10^{3}W}=0.3575kW$ (S4)

**Electrolytic cell cost:**

According to the E4tech/Element Energy report [2] and DOE's H2A analysis of central grid electrolysis.^3^ Due to the absence of precious metals in the PEC electrolytic cell, adjustments were made to the anode components, with the anode cost based on the amount of target material consumed in the preparation of TiO_2_ thin films. The unit cost of the PEC electrolytic cell stack components is 519.5 USD kW‧h^-1^, while the comparative electrocatalytic electrolytic cell model, based on previously reported models, has a unit cost of 550 USD kW‧h^-1^ [4]. The reference current density is 400 milliamps per square centimeter, and the voltage is 1.75 volts. The installation factor for capital investment is 1.12 (referring to the US Department of Energy H2A basic model). The total cost of the electrolytic cell is calculated as follows:

$Electrolytic\text{ }cell\text{ }cost=\frac{519.5 \$ kW^{-1}}{1000\frac{W}{kW}}\times\frac{400 mA\cdot cm^{-2}}{1000\frac{mA}{A}}\times1.75V\times\frac{27.5m^{2}}{0.0001\frac{m^{2}}{cm^{2}}}\times1.12=\$112004.2$ (S5)

Considering the capital recovery rate (CRF) based on a 5% discount rate (represented as i in the following equation), with a validity period of 20 years:

$CRF=\frac{i\left(1+i\right)^{year}}{i\left(1+i\right)^{year}-1}=0.0802$ (S6)

With 350 days of operation per year and a production rate of 1 kg day^-1^ of Li, the cost of an electrolytic cell per kilogram of Li is:

$Electrolytic\text{ }cell\text{ }cost=\$112004.2\times\frac{0.0802}{350 days}\times\frac{1}{1 kg days}=25.665 \$ kg\text{-}Li^{-1}$ (S7)

**Balance of plant (BoP) cost:**

Assuming that the BoP cost is 35% of the total cost of the electrolytic cell system [3, 7]:

$BoP=\frac{25.665 \$ kg\text{-}Li^{-1}}{65\%}\times35\%=13.82 \$ kg\text{-}Li^{-1}$ (S8)

**Electricity cost:**

The electricity bill is calculated based on electricity demand and a price of 0.03 $ kW‧h^–1^, which is based on the target announced by DOE [9, 10]

$Electricity\text{ }cost=0.3575kW\times24h\times\frac{\$0.03}{kWh}\times\frac{1}{1 kg day^{-1}}=0.2574 \$\text{ }kg-Li^{-1}$ (S9)

**Maintenance:**

Assuming the maintenance cost is 2.5% of the annual capital cost of the electrolytic cell [3, 7]:

$Maintenance=\$ 112004.2\times\frac{2.5\%}{year}\times\frac{year}{350 days}\times\frac{1}{1 kg day^{-1}}=8 \$\text{ }kg\text{-}Li^{-1}$ (S10)
